# Detrimental effects of physical inactivity on peripheral and brain vasculature in humans: Insights into mechanisms, long-term health consequences and protective strategies

**DOI:** 10.3389/fphys.2022.998380

**Published:** 2022-09-27

**Authors:** Alessio Daniele, Samuel J. E. Lucas, Catarina Rendeiro

**Affiliations:** ^1^ School of Sport, Exercise and Rehabilitation Sciences, University of Birmingham, Birmingham, United Kingdom; ^2^ Centre for Human Brain Health, University of Birmingham, Birmingham, United Kingdom

**Keywords:** sitting, sedentary, inactivity, vascular, cerebrovascular, endothelial

## Abstract

The growing prevalence of physical inactivity in the population highlights the urgent need for a more comprehensive understanding of how sedentary behaviour affects health, the mechanisms involved and what strategies are effective in counteracting its negative effects. Physical inactivity is an independent risk factor for different pathologies including atherosclerosis, hypertension and cardiovascular disease. It is known to progressively lead to reduced life expectancy and quality of life, and it is the fourth leading risk factor for mortality worldwide. Recent evidence indicates that uninterrupted prolonged sitting and short-term inactivity periods impair endothelial function (measured by flow-mediated dilation) and induce arterial structural alterations, predominantly in the lower body vasculature. Similar effects may occur in the cerebral vasculature, with recent evidence showing impairments in cerebral blood flow following prolonged sitting. The precise molecular and physiological mechanisms underlying inactivity-induced vascular dysfunction in humans are yet to be fully established, although evidence to date indicates that it may involve modulation of shear stress, inflammatory and vascular biomarkers. Despite the steady increase in sedentarism in our societies, only a few intervention strategies have been investigated for their efficacy in counteracting the associated vascular impairments. The current review provides a comprehensive overview of the evidence linking acute and short-term physical inactivity to detrimental effects on peripheral, central and cerebral vascular health in humans. We further examine the underlying molecular and physiological mechanisms and attempt to link these to long-term consequences for cardiovascular health. Finally, we summarize and discuss the efficacy of lifestyle interventions in offsetting the negative consequences of physical inactivity.

## 1 Introduction

Over the past few decades, the increasing prevalence of physical inactivity has become one of the most critical public health problems of our society (Dumith et al., 2011; Harvey et al., 2013). According to the World Health Organization recommended physical activity guidelines (Bull et al., 2020), physical inactivity refers to an insufficient level of moderate (at least 150 min per week) or vigorous intensity aerobic physical activity (at least 75 min per week). Physical inactivity is recognized as an independent risk factor for different pathologies including atherosclerosis (Bertoni et al., 2009), hypertension (Huai et al., 2013), cardiovascular disease (CVD) (Fletcher et al., 1996), type 2 diabetes (Admiraal et al., 2011), and breast (Pizot et al., 2016) and colon cancer (Liu et al., 2016). Globally, it is estimated that 1 in 4 adults and over 80% of adolescents are physically inactive (Guthold et al., 2018; Guthold et al., 2020). However, in many geographical areas, such as Latin America and Caribbean, and high-income Western countries, the average inactivity level is above one-third of the adult population (Guthold et al., 2018). This is a public health concern given that physical inactivity is the fourth leading risk factor for mortality (British Heart 2017) accounting for more than five million deaths per year (Ekelund et al., 2016). Furthermore, physical inactivity has been shown to have detrimental effects on brain function in children (Chaddock et al., 2011) and older adults (Arnardottir et al., 2016) and be associated with an increased risk of dementia (Laurin et al., 2001; Hamer and Chida 2009; Kivimäki et al., 2019).

Importantly, being physically active may not be sufficient to prevent the detrimental effects on health. There is accumulating evidence from both epidemiological and observational studies showing that sedentary behaviour *per se* is a major modifiable risk factor for chronic disease and is predictive of cardiovascular and all-cause mortality (Ekelund et al., 2016; Carter et al., 2017). Sedentary behaviour is characterized by any waking behaviour (e.g., TV viewing, computer use) in a sitting, reclining or lying position in which the energy expenditure is ≤ 1.5 metabolic equivalents (METs) (Tremblay et al., 2017). Numerous studies indicate that links between sedentary time and mortality may be apparent even among those who are physically active (Carter et al., 2017; Patterson et al., 2018). Notably, others report that physical activity (more than 60 min per day of moderate intensity, or more than 150 min per week of moderate-to-vigorous intensity) can be effective in attenuating or even eliminating the increased mortality risks associated with sedentary time (Ekelund et al., 2016; Stamatakis et al., 2019). The prevalence of sedentary behaviour has increased drastically in western countries within the last 50 years (Church et al., 2011; Archer et al., 2013), with young adults spending approximately 8.5 h daily in sedentary activities (Staiano et al., 2018) most of which are work-related (approx. 60% of total daily sitting time) (Clemes et al., 2014; Kazi et al., 2014). This is more accentuated in older adults, with reports showing that individuals over 60 years old spend more than 9.5 h per day being sedentary (Matthews et al., 2008; Sagelv et al., 2019). This is particularly relevant given that spending more than 9.5 h per day being sedentary increases the risk of all-cause mortality by approximately 30% (i.e., hazard ratio: ∼1.3) (Ekelund et al., 2019). Moreover, to aggravate the already critical situation, future predictions estimated that the amount of time spent in sedentary behaviour will increase in the foreseeable future (Ng and Popkin 2012).

Importantly, randomized controlled human trials show that even acute bouts of uninterrupted sitting can have adverse effects on peripheral (Thosar et al., 2014; Thosar et al., 2015a; Thosar et al., 2015b; Restaino et al., 2015; Morishima et al., 2016; Restaino et al., 2016; Morishima et al., 2017; Vranish et al., 2018), central (Dempsey et al., 2016a; Dempsey et al., 2016b; Credeur et al., 2019) and cerebral vascular health (Carter et al., 2018) as well as on glucose metabolism (Dunstan et al., 2012; Bailey and Locke 2015). It is likely that repeated transient impairments in vascular and metabolic function due to acute sitting may contribute to the well-known, long-term detrimental effects on cardiovascular (Padilla and Fadel 2017), metabolic (Kim et al., 2018), and brain health (Falck et al., 2017; Siddarth et al., 2018). However, the underlying mechanisms linking acute/short-term effects of sitting and long-term consequences for human health are not well understood. Uncovering such mechanistic underpinnings will be key to optimize existent strategies (e.g., standing, walking) and foster new alternative strategies (e.g., dietary approaches) that can minimize or counteract the detrimental effects of acute sedentariness in humans.

In this review, we summarize the evidence supporting the impact of acute and short-term physical inactivity on peripheral and cerebral vascular function in humans. We further discuss the potential molecular and physiological mechanisms that underlie such detrimental effects and attempt to link these to long-term consequences for health. Additionally, we summarize and discuss interventions that are efficacious in offsetting the immediate negative consequences of inactivity.

## 2 Acute effects of physical inactivity on vascular function in humans

### 2.1 Endothelial function

Studies examining the acute effects of physical inactivity on human vascular health have been primarily focused on models of uninterrupted sitting, lasting from 10 min to 7 h, in young healthy individuals, middle-aged overweight/obese adults, adults with type 2 diabetes, and women with polycystic ovary syndrome (PCOS) (Table 1). Restaino et al. (2015) showed that prolonged sitting (lasting 6 h) resulted in a decline in endothelial function, measured by flow-mediated dilation (FMD), in the popliteal artery in the lower limbs in young healthy men. Conversely, FMD of the brachial artery in the upper limbs remained unaffected by sitting (Restaino et al., 2015), which is consistent with what was observed in similar studies (Thosar et al., 2014; Carter et al., 2017).

**TABLE 1:** The acute effects of prolonged sitting on vascular health biomarkers.

Outcome measures						
References	Population (N)	Artery	Sitting time (h)	%FMD	Shear rate/stress	Other
Alansare et al. (2020)	Early middle-aged at risk (25)		>7			SBP: ↑; DBP: ↑; MAP: ↑; PWV: ↑
Ballard et al. (2017)	Young healthy (11)	SFA	3	↓	↓	ET-1: ↔
Caldwell et al. (2020)	Young healthy (26)	SFA	2	↔	↓	ET-1: ↔; PWV: ↔
Carter et al. (2017)	Young healthy (10)	BA	∼1.5	↔	↔	
Carter et al. (2019)	Young healthy (15)	SFA	4	↔	↔	
Cho et al. (2020)	Young healthy (12)	PA - BA	4	BA: ↓	PA: ↔	
Climie et al. (2018)	Late middle-aged at risk (19)	SFA - BA	5	SFA: ↓; BA: ↔	SFA: ↔; BA: ↔	ET-1: ↔
Credeur et al. (2019)	Young (overweight/obese) (20)	PTA	3	↔	↓	Diameter: ↓; L-FMC: ↔; PWV: ↑
Decker et al. (2021)	Young healthy (26)	CFA	1.5			PLM-induced hyperaemia [LBF_Δpeak_ (men: ↓; women: ↓)]
Evans et al. (2019)	Young healthy (20)		3			PWV: ↑; TSI: ↓
Garten et al. (2019a)	Young healthy (20)	CFA	3		Active: ↔; non-active: ↔	PLM-induced hyperaemia [∆LBF (Active: ↓; non-active: ↓); LBF AUC (Active: ↓; non-active: ↓)]
Garten et al. (2019b)	Young healthy (10)	CFA	3			PLM-induced hyperaemia (∆LBF: ↓; LBF AUC: ↓)
Hartman et al. (2021)	Elderly at risk (24)	SFA	3	↔		
Headid et al. (2020)	Young healthy (12)	PA - BA	2.5	PA: ↓; BA: ↓		PWV: ↔; AIx: ↓; TSI: ↓
Kowalsky et al. (2019)	Late middle-aged at risk (14)		4			PWV: ↔
Kruse et al. (2018)	Early middle-aged (overweight/obese) (13)	PA	4	↓	↓	
Liu et al. (2021)	Young healthy (21)	PA	3	↓	↓	Blood flow: ↓
McManus et al. (2015)	Young healthy girls (9)	SFA	3	↓	↔	
Morishima et al. (2016)	Young healthy (11)	PA	3	↓	↓	
Morishima et al. (2017)	Young healthy (15)	PA	3	↓	↓	
Morishima et al. (2020a)	Young healthy (19)	PA	3	↓	↓	
Morishima et al. (2020b)	Young healthy (19)	PA	3	Active: ↔; non-active: ↓	Active: ↓; non-active: ↓	
O’Brien et al. (2019)	Young healthy (20)	PA	3	Men: ↓; women: ↓	Men: ↓; women: ↓	L-FMC (men: ↓; women: ↓)
O’Brien et al. (2020)	Young healthy (18)	PA	3	↓	↓	
Padilla et al. (2009)	Young healthy (11)	PA	3	↔	↓	
Peddie et al. (2021)	Young healthy (18)	PA	6	↔	↓	Blood flow: ↓
Perdomo et al. (2019)	Middle-aged healthy (15)		0.5			PWV: ↔
Restaino et al. (2015)	Young healthy (11)	PA - BA	6	PA: ↓; BA: ↔	PA: ↓; BA: ↓	
Restaino et al. (2016)	Young healthy (10)	PA	3	↓	↓	
Taylor et al. (2020)	Young at risk (13)	SFA	3.5	↔	↔	
Taylor et al. (2021)	Middle-aged/elderly at risk (24)	SFA	7	↔	↔	ET-1: ↓
Thosar et al. (2014)	Young healthy (12)	SFA - BA	3	SFA: ↓; BA: ↔	SFA: ↓; BA: ↔	
Thosar et al. (2015a)	Young healthy (12)	SFA	3	↓	↓	
Thosar et al. (2015b)	Young healthy (11)	SFA	3	↓	↓	
Tremblay et al. (2019)	Young Healthy (11)	SFA	0.5	↔	↓	PWV: ↔
Vranish et al. (2017)	Young healthy (20)	PA	3	Men: ↓; women ↔	Men: ↓; women ↓	
Vranish et al. (2018)	Young healthy (14)	PA	0.17	↔	↓	

AIx, augmentation index; AUC, area under the curve; BA, brachial artery; CFA, common femoral artery; DBP, diastolic blood pressure; ET-1, endothelin-1; LBF, leg blood flow; L-FMC, low-flow mediated constriction; MAP, mean arterial pressure; PA, popliteal artery; PLM, passive leg movement; PTA, posterior tibial artery; PWV, pulse wave velocity; SBP, systolic blood pressure; SFA, superficial femoral artery; TSI, tissue saturation index.

First introduced in 1992 (Celermajer et al., 1992), FMD is now a widely popular, non-invasive tool for examining nitric oxide (NO)-dependent endothelial function of peripheral conduit arteries (Thijssen et al., 2019). The FMD technique requires the use of an ultrasound device (ideally, high-resolution, and duplex); the protocol consists of inducing a post-ischemic increase in blood flow (and therefore shear stress) that results in a transient increase in diameter (i.e., vasodilation) of the imaged conduit artery (Thijssen et al., 2019). Peripheral conduit arteries such as the brachial (Green et al., 2014) and superficial femoral artery (Kooijman et al., 2008) have been demonstrated to be largely mediated by NO, therefore reflecting endothelium-dependent vascular function. FMD decline reflects endothelial dysfunction, which is typically an early sign of atherosclerosis (Mudau et al., 2012; Thijssen et al., 2019). FMD is affected by sedentary behaviour, particularly, as showed in a recent study, large amount of total daily sitting time has been associated with reductions in FMD (Yamaji et al., 2022). Whilst brachial FMD is reflective of future risk of CVD (decline of 1% in FMD is associated with an increased risk of a future CVD event of up to 13%) (Inaba et al., 2010), lower limb FMD seems to be more closely associated with lower limb atherosclerosis and peripheral arterial disease (PAD) (Heinen et al., 2015). Indeed, in an asymptomatic population, higher sedentary time has been associated with a 22% increased risk of a future diagnosis with PAD (Kulinski et al., 2015).

Although only a limited number of studies have assessed the impact of prolonged sitting on endothelial function on both upper and lower limbs, most have found that sitting affects mainly endothelial function in the lower limb arteries (Thosar et al., 2014; Restaino et al., 2015; Climie et al., 2018). To date, only two studies have shown significant decline in brachial FMD in response to uninterrupted sitting (i.e., 2.5, and 4 h) (Cho et al., 2020; Headid et al., 2020), with one of those two studies showing concomitantly a marked decline in popliteal FMD (Headid et al., 2020). In agreement with Restaino et al. (2015), many others have shown that shorter sitting trials (1–3 h) have detrimental effects on endothelial function not only in the popliteal artery (2.3–4.1% FMD) (Morishima et al., 2016; Restaino et al., 2016; Morishima et al., 2017; Vranish et al., 2017; Morishima et al., 2020a; Morishima et al., 2020b) but also in the upstream superficial femoral artery (2.4–2.7% FMD) in young healthy adults (Thosar et al., 2014; Thosar et al., 2015a; Thosar et al., 2015b; Ballard et al., 2017). In addition, recent studies have found that the common femoral artery is affected by sitting-induced vascular dysfunction, measured by passive leg movement (PLM)-induced hyperaemia (Garten et al., 2019a; Garten et al., 2019b; Decker et al., 2021), an alternative method to assess endothelial function (Gifford and Richardson 2017).

Significant declines in lower limb vascular function, specifically superficial femoral artery (4.1–4.3% FMD), seem to be observed consistently within the first hour of sitting, with no significant further declines during longer periods of sitting (Thosar et al., 2014; Thosar et al., 2015a; Thosar et al., 2015b; Climie et al., 2018). Interestingly, among those studies, only one showed a small recovery in FMD detectable from the second hour of sitting that was maintained up to 5 h (Climie et al., 2018). Despite the majority of the studies indicating that sitting-induced impairments in vascular function occur in the lower limbs, a few studies report no statistical significant differences in FMD in femoral (Carter et al., 2019; Caldwell et al., 2020; Taylor et al., 2020; Hartman et al., 2021; Taylor et al., 2021), popliteal (Padilla et al., 2009; Peddie et al., 2021), and posterior tibial artery (Credeur et al., 2019) in response to 2–7 h of sitting (Table 1).

The level of physical fitness may also play an important role in counteracting sitting-induced vascular dysfunction, but the literature is rather limited and shows discrepancy in results. Specifically, one of these studies has shown that endurance-trained men, contrary to their untrained counterparts, maintained lower limb endothelial function throughout a 3-h sitting period (Morishima et al., 2020a), whereas other studies do not confirm these findings (Garten et al., 2019a; Liu et al., 2021). In this regard, Liu et al. (2021) have shown that higher fitness levels were associated with more pronounced sitting-induced declines in popliteal FMD, despite having higher FMD prior to sitting (Liu et al., 2021). Additionally, another study has found similar sitting-induced declines in lower limb vascular function between trained and untrained individuals (Garten et al., 2019a), although in this study endothelial function was assessed using the PLM technique, and not the gold-standard FMD. As such, it is not clear whether physical fitness is sufficient to provide a protective effect against sitting-induced endothelial dysfunction and this is an area that should be investigated in future studies.

In children, sitting-induced negative consequences on vasculature are remarkably similar. For example, a study in 7–10 year-old girls showed that sitting-induced impairments in endothelial function in the superficial femoral artery may also be apparent within similar time frames (i.e., 3 h) (McManus et al., 2015) and to a similar degree (2.3% FMD) to young healthy adults (Thosar et al., 2014; Thosar et al., 2015a; Thosar et al., 2015b; Ballard et al., 2017). This is relevant considering that children (9–11 years old) spend on average more than 8 h per day in sedentary activities (LeBlanc et al., 2015), while their recommended daily time for being physical active is 60 min (more than twice that of adults) (Gibson-Moore 2019).

The detrimental effects of sitting have been also observed in at-risk populations. In particular, recent studies in sedentary overweight/obese adults have shown FMD declines in both the femoral (4.1%) and popliteal artery (1.5%) in response to 1 and 4 h of sitting, respectively (Climie et al., 2018; Kruse et al., 2018). No changes in brachial FMD were detected in this population (Climie et al., 2018). On the other hand, more recent studies in women with PCOS (Taylor et al., 2020), adults with type 2 diabetes (Taylor et al., 2021) and with increased cardiovascular risk (Hartman et al., 2021), did not detect any sitting-induced decline in FMD in lower limb arteries, specifically the superficial femoral artery (Taylor et al., 2020; Hartman et al., 2021; Taylor et al., 2021).

Furthermore, it remains unclear whether there are sex differences in vascular responses to prolonged sitting, with a few studies comparing the impact of sitting between males and females. Vranish et al. (2017) reported sitting-induced declines in popliteal artery FMD in men, while in pre-menopausal women (assessed during the early follicular phase) vascular function was preserved, likely due to the influence of oestrogen, which is protective of endothelial function (Maturana et al., 2007). However, more recent studies report similar declines in micro and macrovascular function in the popliteal and common femoral artery in both males and females (O’Brien et al., 2019; Decker et al., 2021). This highlights the need to design future studies that can evaluate the sex differences in vascular function during sitting, also taking into consideration menopausal status.

Altogether, these studies indicate that uninterrupted prolonged sitting in children, healthy young adults and at-risk populations affects peripheral conduit arteries by impairing endothelial-dependent vascular function. Particularly, lower limb arteries are the most affected by sitting, while vascular impairments in upper limb arteries are often not observed. This is important to consider, given that lower limb arteries are more prone to circulatory problems such as atherosclerosis (Kröger et al., 1999; Laclaustra et al., 2016) and peripheral arterial disease (Thachil et al., 2011). However, it remains to be established how the severity of vascular deficits changes with sitting time, but findings to date indicate that endothelial dysfunction can be detected after only 1 h of sitting, with additional sitting time not resulting in more severe impairments in both healthy young adults and sedentary overweight/obese middle-aged adults. Currently, it is unclear how the severity of sitting-induced endothelial dysfunction in at-risk populations (e.g., obese individuals) compares with healthy young adults, given that no studies have directly compared these groups within the same human trial. Finally, the impact of sex and fitness in endothelial function post-sitting requires further examination.

### 2.2 Blood flow haemodynamics

In addition to endothelial function, other parameters such as blood flow and shear rate/stress are also modulated by prolonged sitting in both lower and upper limb arteries (Thosar et al., 2014; Morishima et al., 2017; Vranish et al., 2018). Blood flow and shear rate/stress play important roles in the regulation of vascular tone by stimulating the endothelium to release vasoactive factors such as NO (Paniagua et al., 2001). Marked declines in both blood flow and shear rate in the popliteal artery have been reported in response to very short periods of sitting (10 min) in both healthy young men and women (Vranish et al., 2017; Vranish et al., 2018). Interestingly, this reduction occurred within the first minute of sitting and remained consistently lower until the end of the sitting trial (Vranish et al., 2018). Although popliteal FMD was unaffected after 10 min of sitting, hyperaemic blood velocity (area under the curve) was significantly reduced, suggesting that short periods of sitting affect not only resting blood flow and shear rate, but also functional blood flow dynamics (i.e., reactive hyperaemia response). Reactive hyperaemia, a measure of peripheral microvascular function, is the transient increase in blood flow above baseline that occurs following a short period of ischemia due to arterial occlusion (Rosenberry and Nelson 2020). This is important given that reactive hyperaemia is a predictor of future cardiovascular events, and its reduction has been associated with increased risk of mortality and cardiovascular disease (Huang et al., 2007; Philpott and Anderson 2007; Paine et al., 2016).

Similar declines in blood velocity and shear rate are detected in other lower limb arteries, such as posterior tibial and superficial femoral artery, in young healthy adults (Thosar et al., 2014; Thosar et al., 2015a; Thosar et al., 2015b; Younger et al., 2016; Ballard et al., 2017). However, in at-risk populations, there are contradictory reports, with some studies showing sitting-induced reductions in shear rate and/or blood flow in the lower limb arteries (i.e., posterior tibial and popliteal artery) (Kruse et al., 2018; Credeur et al., 2019), whilst others seeing no changes in blood flow and/or shear rate in the superficial femoral artery (Climie et al., 2018; Taylor et al., 2020; Taylor et al., 2021). It is currently unclear whether these differences are of significance. On the other hand, shear rate declines in the brachial artery appear to be less accentuated when exposed to prolonged sitting. In fact, some studies show no significant reductions in shear rate in both healthy (Carter et al., 2017) and at-risk populations (Climie et al., 2018), whilst in young healthy adults, some show significant declines (Restaino et al., 2015) but also no effects on mean shear rate (Thosar et al., 2014) in young healthy individuals.

Similarly, blood flow has also shown to be reduced in the brachial artery (by approximately 42%) following 2 h of sitting (Restaino et al., 2015). This suggests that the contribution of resting blood flow/shear rate to changes in endothelial function after sitting might be different in upper and lower limbs. Overall, declines in blood flow and/or shear rate after sitting seem to be generally paralleled by reductions in FMD, at least in the lower limbs, suggesting that such haemodynamic changes might contribute to sitting-induced endothelial dysfunction.

### 2.3 Arterial stiffness

Recent studies suggest that sitting (3 h) can negatively impact aortic stiffness in young adults as indicated by an increase in pulse wave velocity (PWV) (∼0.35 m⋅s^−1^) (Credeur et al., 2019; Evans et al., 2019), which is the gold standard measure of arterial stiffness (Townsend 2016). Likewise, an increase in PWV has also been observed in at-risk middle-aged adults during a simulated seated workday (total sitting time: >7 h) (Alansare et al., 2020). These sitting-induced effects on PWV have been reported in young healthy adults (Evans et al., 2019), overweight/obese adults (Credeur et al., 2019) and middle-aged at-risk adults (overweight/obese, hypertensive) (Alansare et al., 2020), highlighting that sitting can have broader effects on vascular health beyond lower limb arterial function. However, similar to FMD findings, there are inconsistencies across the literature with some studies reporting that PWV remains unaltered following uninterrupted prolonged sitting (Kowalsky et al., 2019; Perdomo et al., 2019; Tremblay et al., 2019; Caldwell et al., 2020; Headid et al., 2020). Nevertheless, while such studies fail to show any acute change in PWV that reached the clinically significant threshold of 1 m⋅s^−1^ (corresponding to an increased risk of 14% and 15% in cardiovascular events and all-cause mortality, respectively) (Vlachopoulos et al., 2010), it is plausible to consider that repeated prolonged periods of sitting may aggravate aortic stiffness in the long-term. Indeed, numerous studies reported a positive association between sedentary time and arterial stiffness (Huynh et al., 2014; Horta et al., 2015; Ahmadi-Abhari et al., 2017). Also, this is particularly relevant given that increases in arterial stiffness are associated with higher risk of hypertension (Safar et al., 2018), cardiovascular disease (Mitchell et al., 2010), stroke (Mattace-Raso et al., 2006), chronic kidney disease (Oh 2018), and structural brain damage (Palta et al., 2019).

Sitting also induces increases in oscillatory shear index (OSI) in the brachial artery (Thosar et al., 2014), whilst resulting in either a reduction or no effect in the lower limb arteries (McManus et al., 2015; Garten et al., 2019a; Credeur et al., 2019). OSI is a haemodynamic parameter that reflects fluctuations in blood flow (Zhang et al., 2014), and is negatively correlated with wall shear stress (Fytanidis et al., 2014). Numerous studies have reported that a high OSI value may contribute to the development of atherosclerotic disease and arterial wall thickening (Goubergrits et al., 2008; Wen et al., 2010; Fytanidis et al., 2014; Meirson et al., 2015), which is shown to correlate with arterial stiffness (Gómez-Marcos et al., 2011; Suceava et al., 2013). Therefore, while most studies that have investigated upper limb arteries have not shown impairments in endothelial function after sitting (e.g., Thosar et al., 2014), the changes in OSI reported to date suggest that upper limb arteries may still be susceptible to atherosclerosis due to prolonged sitting. More studies are needed to clearly establish the short and long-term effects of sitting on markers of vascular stiffness.

### 2.4 Arterial blood pressure

Prolonged sitting has also been shown to have detrimental effects on blood pressure (BP). However, the literature remains unclear with some studies reporting no sitting-induced variations in BP (e.g., Restaino et al., 2016; Carter et al., 2019; Headid et al., 2020), whilst several studies have detected significant increases in either systolic (SBP), diastolic (DBP), or mean arterial pressure (MAP) in response to short bouts of sitting (i.e., 3–7 h) (e.g., O’Brien et al., 2019; Morishima et al., 2020a; Morishima et al., 2020b). For example, Younger et al. (2016) have reported that 5 h of sitting leads to increases in MAP of approximately 5 mmHg, in healthy young adults. In addition to this, Dempsey et al. (2016a) showed increases in SBP (10 mmHg) and DBP (5 mmHg) after 7 h of uninterrupted sitting in late middle-aged/elderly adults (∼60 years old) with cardiovascular risk factors. Effectively, these BP changes reached clinically relevant pre-hypertensive range (above 130/80), which are known to be associated with an increased risk of developing hypertension (Chobanian et al., 2003) and future cardiovascular events (Huang et al., 2013). Importantly, data pooled from 4 randomized controlled trials indicate that sitting might increase BP in a dose-response manner and that hypertensive individuals might be more susceptible to sitting-induced increases in BP (∆SBP = 6 mmHg; ∆DBP = 3 mmHg) than normotensives (∆SBP = 5; ∆DBP = 2 mmHg), as observed following 6 h of sitting (Dempsey et al., 2018). This is key, as acute increases in BP during sitting might put this already vulnerable population at even higher risk of future cardiovascular events (Luo et al., 2020).

In summary, uninterrupted prolonged sitting induces detrimental effects on endothelial function in the peripheral vasculature, increases arterial stiffness and raises blood pressure, as well as modulates blood flow and shear rate/stress in both healthy and middle-aged obese/overweight populations. The lower limb arteries appear to be much more susceptible to sitting-induced vascular impairments compared to upper body arteries (e.g., brachial artery), although only a few studies have addressed this question specifically.

## 3 Short-term effects of physical inactivity in the peripheral vasculature

**FIGURE 1:**
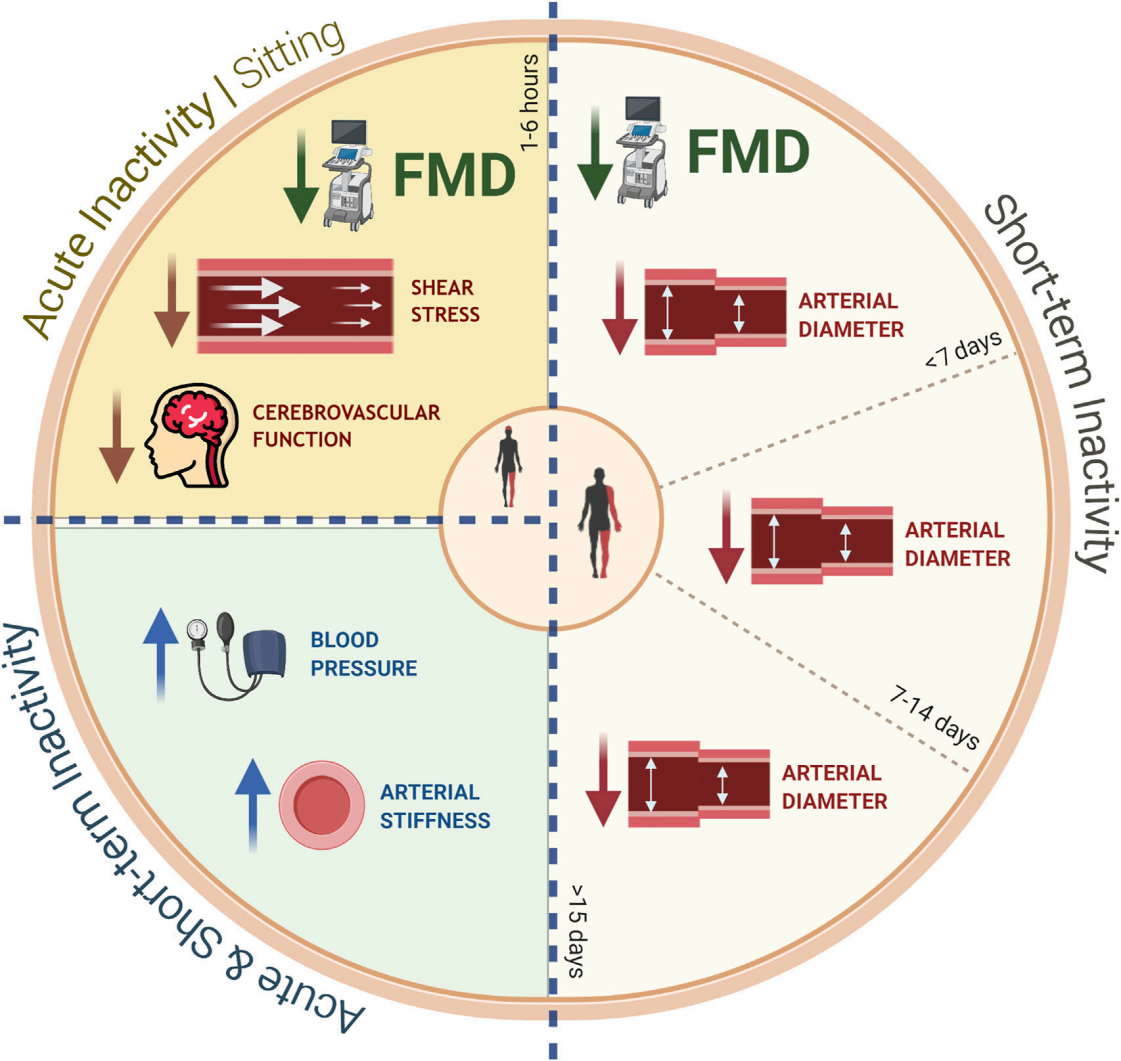
Summary of the negative effects of both acute (sitting) and short-term (e.g., step-reduction, limb immobilization, bed rest) inactivity protocols on peripheral vascular health in humans: sitting for up to 6 h results in a reduction in FMD, shear stress/rate, and cerebrovascular function; short-term reductions in physical activity of up to 15 days (or more) result in declines in arterial diameter. All inactivity protocols (acute and short-term) have been reported to increase blood pressure and arterial stiffness. *FMD: Flow-mediated dilation*.

The literature investigating the effects of physical inactivity on human vascular function over short periods of time is currently limited, but there is evidence to indicate that periods of reduced/abolished physical activity ranging from 1 to 8 weeks can have detrimental consequences (Thijssen et al., 2010) (Figure 1) (Table 2). In particular, step-reduction models have shown marked declines in the popliteal artery FMD response (approx. 3.5%) after 5 days of reduced activity (from >12,000 to <4,000 steps per day) in healthy active men (Boyle et al., 2013; Teixeira et al., 2017). Whilst no changes in FMD were detected in the brachial artery, a significant reduction in resting arterial diameter was reported (0.23 mm) (Boyle et al., 2013). Interestingly, when in young healthy adults the daily step count was further reduced (from >12,000 to approx. 2,500) and for a longer period of time (i.e., 14 days) there were observed reductions in brachial FMD (by 1.8%) while the diameter remained unaltered (Bowden Davies et al., 2021). This may suggest that intensity and duration of the physical inactivity protocol play an important role in mediating inactivity-induced vascular dysfunction. To date, the effects of step-reduction on vascular health in older adults or other at-risk populations have not been investigated. This would be particularly important future work given that in the United Kingdom, older adults perform an average of 4,000–6,500 steps per day (Harris et al., 2009; Davis et al., 2011; Ludwig et al., 2018), with over 80 year-olds performing less than 3,000 steps daily (Davis et al., 2011). Additionally, increases in circulating levels of endothelial microparticles (EMPs) have also been observed (Boyle et al., 2013). EMPs are small membrane-enclosed vesicles released in response to endothelial cell injury/apoptosis and represent an accurate biological marker of endothelial dysfunction (Deng et al., 2016). Indeed, direct relationships between microparticle concentrations and clinical manifestations of cardiovascular diseases, such as atherosclerosis (Paudel et al., 2016), coronary artery disease (França et al., 2015) and myocardial infarction (Deng et al., 2016), have been observed.

**TABLE 2:** The effects of short-term inactivity on vascular health biomarkers.

Outcome measures							
References	Population (N)	Artery	Inactivity model (days)	%FMD	Shear rate/stress	Diameter	Other
Birk et al. (2013)	Young healthy (13)	BA	Forearm immobilization (8)	↔		↔	
Bleeker et al. (2005)	Young healthy (16)	SFA - CFA - BA- CCA	Bed rest (52)	SFA: ↑		SFA: ↓; CFA: ↓; BA: ↓; CCA: ↔	
Bowden Davies et al. (2021)	Young healthy (28)	BA	Step reduction (14)	↓		↔	
Boyle et al. (2013)	Young healthy (11)	PA - BA	Step reduction (5)	PA: ↓; BA: ↔		PA: ↔; BA: ↓	EMPs: ↑
Cohen et al. (2021)	Young healthy (31)	SFA	Leg immobilization (14)	↔	↔	↓	PWV: ↔
Hamburg et al. (2007)	Young healthy (22)	BA	Bed rest (5)	↔		↓	
Hyldahl et al. (2021)	Young healthy (21)	CFA	Leg immobilization (10)			↓	PLM-induced hyperaemia: ↓; VEGF: ↓; ET-1: ↔
Nosova et al. (2014)	Young healthy (5)	SFA - BA	Bed rest (5)	SFA: ↓; BA: ↓	SFA: ↔; BA: ↔	SFA: ↔; BA: ↔	PWV: ↔; AIx: ↑
Rakobowchuk et al. (2011)	Young healthy (15)	PA - CFA - CCA	Leg immobilization (12)	PA: ↑	PA: ↑; CFA: ↑	PA: ↓; CFA: ↓; CCA: ↔	
Sugawara et al. (2004)	Young healthy (8)	CFA	Leg immobilization (7)		↔	↓	IMT: ↔; Vascular conductance: ↓
Teixeira et al. (2017)	Young healthy (13)	PA	Step reduction (5)	↓		↔	

AIx, augmentation index; BA, brachial artery; CCA, common carotid artery; CFA, common femoral artery; EMPs, endothelial microparticles; ET-1, endothelin-1; IMT, intima-media thickness; PA, popliteal artery; PLM, passive leg movement; PWV, pulse wave velocity; SFA, superficial femoral artery; VEGF, vascular endothelial growth factor.

Prolonged bed rest is an alternative method of restricting physical activity in humans and has been widely used in research in recent years to evaluate its impact on human metabolic and vascular function (Jost 2008; Hargens and Vico 2016). In contrast with step-reduction models, in which the main activity (i.e., walking) has an energy expenditure of approx. 3 METs, bed rest abolishes any form of activity resulting in an energy expenditure of only 1 MET (Mendes et al., 2018), corresponding to resting metabolic rate (Jetté et al., 1990). Prolonged bed rest is widely prevalent during hospitalization, however, although traditionally recommended as a primary treatment for many medical conditions, prolonged bed rest can be considered as a deleterious form of therapy (Allen et al., 1999). Nosova et al. (2014) have shown that a 5-day bed rest intervention induces a significant decline in FMD in brachial and superficial femoral arteries (approx. 2% in both), an increase in DBP (approx. 4 mmHg) and an increase in central augmentation index (9%, as an indirect measure of arterial stiffness) in young healthy individuals (Nosova et al., 2014). A similar period of bed rest was shown to result in further reductions in the diameter of the brachial artery, and an increase in SBP (approx. 7 mmHg), although there were no significant changes in blood flow, blood velocity and FMD detected in this study (Hamburg et al., 2007). Impairments in microvascular function, as shown by decreased reactive hyperaemia, were also detected in both upper and lower limbs (Hamburg et al., 2007). For example, the aforementioned study by Hamburg and colleagues also observed a reduction in peak hyperaemia blood flow, which is traditionally used as a surrogate measure of resistance vessel remodelling and may indicate the presence of inward remodelling of resistance arteries (Thijssen et al., 2011). This finding is consistent with the inward remodelling of the brachial conduit artery which was also reported, as indicated by the reduction in brachial artery diameter (Hamburg et al., 2007). This aspect is clinically relevant given that structural inward remodelling of resistance vessels is associated with an increased risk of future cardiovascular events (Brown et al., 2018).

Longer bed rest protocols, as expected, seem to induce more severe effects on the vasculature. For example, Bleeker et al. (2005) reported that 52 days of horizontal bed rest resulted in diameter reductions of the brachial (5%), superficial (16%) and common femoral artery (17%), indicating that arterial inward remodelling may have occurred as a consequence of short-term inactivity. However, no alterations in BP, and in baseline blood flow in all examined arteries were observed. Interestingly, the reduction in diameter observed in the superficial femoral artery was paralleled by an increase in FMD and endothelium-independent dilation, possibly due to increased smooth muscle sensitivity to vasodilators (e.g., NO), as suggested by the authors (Bleeker et al., 2005), or simply because of the inverse relationship between FMD and baseline diameter (Silber et al., 2001). However, a similar study using the same model found functional impairments in the popliteal vein whilst no significant alterations in diameter were observed (van Duijnhoven et al., 2008).

Localized physical inactivity in the lower limbs have also been achieved experimentally by using unilateral leg casting. Similar to bed rest, this method is used in medical practice, but mainly as a primary treatment for immobilizing a limb after an acute hard and/or soft-tissue injury. To investigate injury-related vascular impact, Sugawara et al. (2004) evaluated the effects of short-term (i.e., 7 days) cast immobilization of the right lower limb (left leg used as an internal control) in healthy young men. They reported significant reductions in resting blood flow, lumen diameter and vascular conductance in the common femoral artery of the immobilized leg; however, there were no significant changes in BP and femoral artery intima-media thickness. Similarly, the study of Rakobowchuk et al. (2011) involved a similar immobilization protocol for a longer period (i.e., 12 days) and showed a decline in diameter, and an increase in shear rate and FMD in the popliteal artery of the immobilized leg in comparison to the control leg. Interestingly, the common femoral artery reported a decline in arterial diameter in both legs, likely driven by an overall reduction in physical activity (Rakobowchuk et al., 2011). These reductions in diameter observed in lower limb arteries are consistent with the findings of a more recent study that used a 14-day leg immobilization protocol (Cohen et al., 2021). Specifically, a reduction in femoral artery diameter was observed whilst FMD, shear rate, and PWV remained unaltered (Cohen et al., 2021). This type of intervention has also been shown to induce a decrease in vascular function (measured by PLM technique), and vascular endothelial growth factor (VEGF) (Hyldahl et al., 2021), which plays an important role in maintaining vascular function (Koyama et al., 2002).

Interestingly, although localized inactivity of lower limbs appears to induce localized vascular impairments, that does not seem to be the case for upper limb arteries. For example, a study investigating specifically the immobilization of the upper limbs reported that brachial artery diameter, FMD and endothelium-independent vasodilation did not change significantly in healthy individuals in response to an 8-day intervention (Birk et al., 2013). However, Birk and colleagues did observe a decline in peak blood flow (Birk et al., 2013), possibly indicating vascular inward remodelling of forearm resistance arteries (Thijssen et al., 2011; Birk et al., 2013). This finding might also be an indication that inactivity-induced vascular remodelling in large conduit arteries and resistance vessels have a different time-course (reviewed in Thijssen et al., 2011). Collectively, these studies suggest that the lower body arteries are more susceptible to localized acute and short-term inactivity than the upper body arteries.

There are other models that reflect local or systemic physical inactivity such as unilateral lower limb suspension, dry immersion, water immersion, and head-down tilt bed rest. However, these inactivity protocols are used primarily to investigate the physiological consequences of microgravity conditions that are relevant for human space travel (Hackney and Ploutz-Snyder 2012; Tesch et al., 2016; Watenpaugh 2016; Tomilovskaya et al., 2019; Pandiarajan and Hargens 2020), and do not represent relevant earth-based sedentary activity, and therefore are out of the scope of this review.

In summary, there is evidence to indicate that short-term inactivity has a detrimental impact on peripheral and central vascular health. Whilst short periods of step-reduction and bed rest induce impairments in peripheral vascular function (as seen in FMD decline), longer systemic and lower limb inactivity protocols seem to impact arterial structure (vascular inward remodelling), potentially reflecting the time-course of vascular adaptations to inactivity. However, most studies have focused on young healthy adults, making the translation of these observations to other populations difficult. Furthermore, upper limb arteries seem to be more resistant than lower body arteries in response to short-term inactivity, particularly following local inactivity protocols, which is consistent with observations from acute sitting studies.

## 4 Effects of physical inactivity in the cerebral vasculature

The body of research looking at the impact of physical inactivity on the cerebral vasculature is currently very limited, mostly focused on the acute effects of prolonged sitting. A recent study in young healthy desk workers reported a decrease in blood velocity in the middle cerebral artery (MCAv; –2.9 cm⋅s^−1^), measured by transcranial Doppler ultrasound, and impaired cerebral autoregulation following 6 h of uninterrupted sitting. However, other cerebrovascular functional measures were not altered (i.e., cerebrovascular CO_2_ reactivity [5% CO_2_]) nor was there a measurable effect on cognitive performance (Carter et al., 2021). Prior studies in the same population using shorter sitting protocols (i.e., 4 h) have reported a reduction in MCAv (–1.4 cm⋅s^−1^), whilst cerebrovascular CO_2_ reactivity (5% CO_2_) and cerebral autoregulation remained unaltered (Carter et al., 2018). Furthermore, in young healthy adults, 3 h of sitting did not result in changes in prefrontal cortex haemodynamics (i.e., total haemoglobin concentration, and tissue saturation index) and executive function (Stoner et al., 2019). In addition, Wheeler et al. (2019) reported larger reductions in MCAv in response to 2 (approx. 6 cm⋅s^−1^) and 4 h (approx. 8 cm⋅s^−1^) of sitting in overweight/obese older adults (Wheeler et al., 2019), which may indicate that age or health status (e.g., endothelial dysfunction) can play a role in cerebrovascular responses to inactivity (Iadecola and Davisson 2008; Stefanidis et al., 2019). Conversely, in response to a much shorter period of sitting (i.e., 30 min), no significant changes in MCAv were observed in middle-aged overweight adults (Perdomo et al., 2019).

Further studies in older adults also report a significant increase in cerebrovascular resistance (Maasakkers et al., 2020), which has been associated with mild cognitive impairment and Alzheimer’s disease (Yew et al., 2017; Nation 2018). Only a limited number of studies have investigated the impact of longer periods of inactivity (e.g., lower limb immobilization, bed rest) in blood flow to the brain, with studies to date reporting no changes in carotid blood velocity, blood flow, arterial compliance and arterial diameter in young healthy individuals (Bleeker et al., 2005; Rakobowchuk et al., 2011).

In summary, the current literature on the impact of short-term periods of inactivity on cerebrovascular function is very limited, yet this is an important area requiring attention, given the evidence suggesting that chronic cerebral hypoperfusion is a key contributing factor to the pathogenesis of dementia (Roher et al., 2012; Wolters et al., 2017; Park et al., 2019) and the epidemiological links between sedentary behaviour and incidence of dementia (Yan et al., 2020). Preventing transient and/or permanent declines in cerebral blood flow by reducing physical inactivity, may be key for delaying the onset of dementia.

## 5 Mechanisms underlying inactivity-induced impairments in vascular function

### 5.1 Vascular biomarkers

**FIGURE 2:**
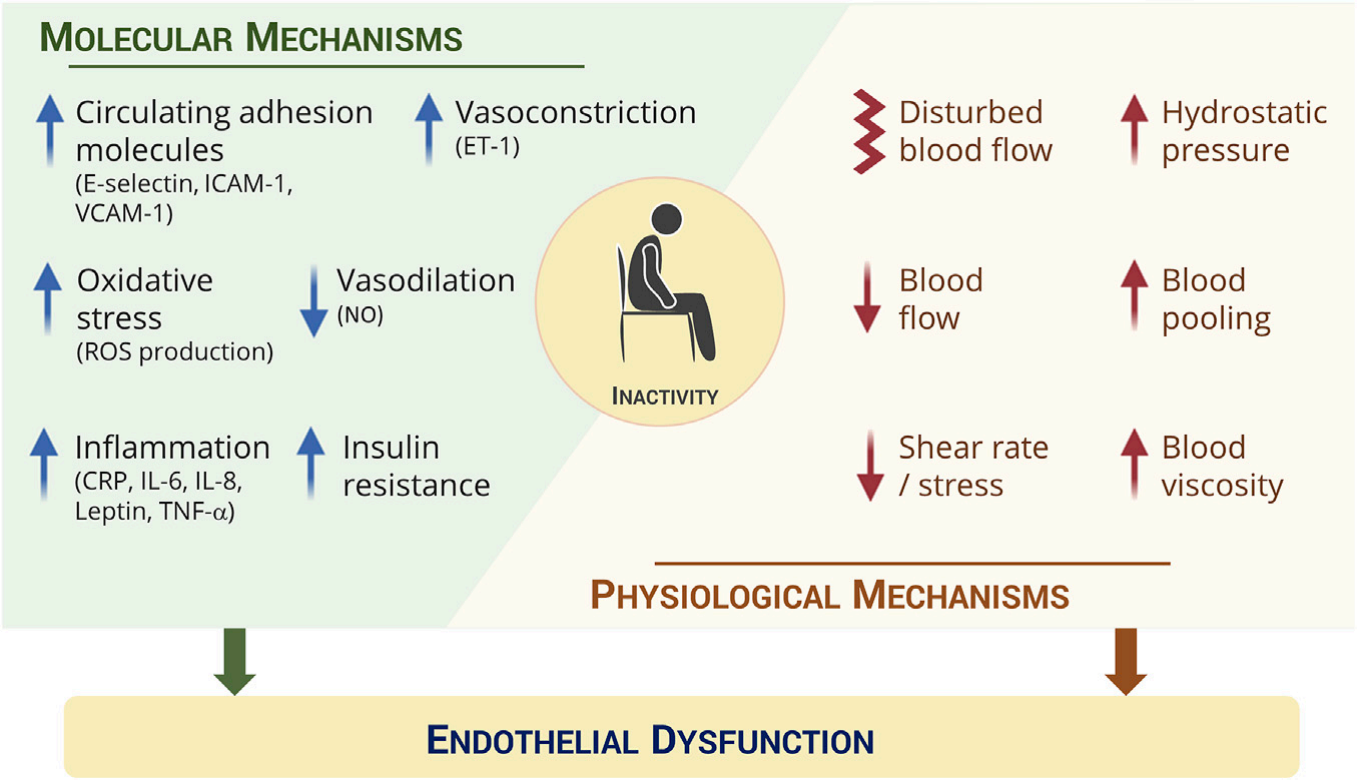
Proposed underpinning mechanisms by which physical inactivity induces endothelial dysfunction in humans include modulation of *i)* molecular components associated with vasodilation/vasoconstriction, vascular adhesion, inflammation, oxidative stress and insulin resistance, as well as *ii)* physiological components, mainly in the lower limbs, such as declines in blood flow and shear stress/rate and increases in hydrostatic pressure, blood pooling and viscosity. *CRP: C-reactive protein; ET-1: Endothelin-1; ICAM-1: Intercellular adhesion molecule-1; IL-6: Interleukin-6; IL-8: Interleukin-8; NO: Nitric oxide; ROS: Reactive oxygen species; TNF-α: Tumor necrosis factor alpha; VCAM-1: Vascular cell adhesion molecule-1*.

The molecular mechanisms underlying the effects on the vasculature of prolonged uninterrupted sitting, remain largely unexplored. It is well-established that endothelial function (measured by FMD) is dependent on a balance between key vasoconstrictors, such as endothelin-1 (ET-1), and vasodilators, such as NO (Sanders et al., 2000; Bourque et al., 2011) (Figure 2). NO is produced by endothelial nitric oxide synthase (eNOS) in response to changes in shear stress (Ballermann et al., 1998; Davies 2009; Sriram et al., 2016) and plays a role in preventing arterial stiffness and platelet aggregation (Sugawara et al., 2007; Sandoo et al., 2010). Currently, there is limited evidence for the role of NO on physical inactivity-induced vascular dysfunction in humans. Climie et al. (2018) reported no changes in levels of circulating nitrate and nitrite after 5 h of sitting in sedentary overweight/obese adults, despite significant declines in femoral FMD. In agreement with this, no changes in plasma levels of arginine and its related metabolites (indirect indicators of NO status) were observed after 3 h of sitting in healthy men (Ballard et al., 2017). Although acute periods of prolonged sitting do not seem to elicit changes in NO status, there are studies reporting significant differences in circulating NO metabolites (e.g., nitrite, nitrate) between sedentary and non-sedentary older adults (Bjork et al., 2012; Carmeli et al., 2015). However, the quantification of nitrite and nitrate in circulation may not be an exact reflection of eNOS activity or NO production (Lauer et al., 2001), thus caution should be taken when these biomarkers are considered for assessing the impact of inactivity on NO levels. Instead, nitrite rather than nitrate may be a more robust measure of endogenous NO production (Lauer et al., 2001). Consistent with human studies, animal studies report lower concentrations of plasma NO and expression of eNOS in sedentary mice compared to their active age-matched counterparts (Suvorava et al., 2004; Song et al., 2009; Kang et al., 2019). Similarly, 14 days of hindlimb unweighting in mice resulted in a reduction of eNOS expression in soleus-feed arteries and this was accompanied by impairments of endothelial vasodilatory function (Jasperse et al., 1999; Woodman et al., 2001). This indicates that NO might play a role in the long-term effects of physical inactivity but does not necessarily explain the more acute and transient effects on endothelial function.

Vascular smooth muscle sensitivity to NO may also play an important role in maintaining endothelial function following long periods of inactivity (Bleeker et al., 2005). For example, Jasperse et al. (1999) showed an increased endothelium-independent vasodilation in soleus-feed arteries in response to administration of sodium nitroprusside, a NO donor, following 14 days of hindlimb unweighting in male rats. This enhanced response during physical deconditioning indicates an increase in NO sensitivity by endothelial smooth muscle cells (Jasperse et al., 1999), which is likely a compensatory mechanism in response to chronic reduction of shear stress and NO bioavailability.

Circulating levels of ET-1 have been shown to increase after prolonged sitting, along with significant declines in femoral FMD and shear rate in humans (Ballard et al., 2017). For example, ET-1 concentrations after 5 h of sitting were significantly higher than sitting with intermittent resistance activity and were found to be significantly correlated with blood flow and shear rate, although not with FMD (Climie et al., 2018). The authors suggest that in sedentary populations, interactions between blood flow, shear stress, and ET-1 may contribute to sitting-induced impairment in arterial function, but additional studies are needed to establish a direct relationship with endothelial function. Importantly, increases in levels of ET-1 have been associated with reduced eNOS expression (Rosiansky-Sultan et al., 2006) and impairments in FMD (Nishiyama et al., 2017), as well as elevations in inflammatory markers (i.e., serum interleukin-6), blood pressure and PWV (du Plooy et al., 2015). Furthermore, increased concentrations of ET-1 appear to play a key role in the pathogenesis of hypertension (Schiffrin 2001) and atherosclerosis (Dashwood and Tsui 2002).

Circulating adhesion molecules, such as intercellular (ICAM) and vascular cell adhesion molecules (VCAM), have been reported to be higher in physically inactive women (Mora et al., 2006) and sedentary hypertensive men (DeSouza et al., 1997), and positively associated with TV viewing time in children (Gabel et al., 2016) and adolescents (Martinez-Gomez et al., 2012). Chronic changes in circulating adhesion molecules may be clinically relevant molecular biomarkers for atherosclerosis (Hwang et al., 1997; Blann et al., 1998; Gross et al., 2012; Varona et al., 2019). However, no significant changes have been observed after prolonged sitting or bed rest (Nosova et al., 2014; Climie et al., 2018; Duvivier et al., 2018), so future studies are needed to establish the significance of adhesion molecules to endothelial dysfunction during periods of physical inactivity.

### 5.2 Insulin resistance

Several studies have reported positive associations between sedentary time and insulin resistance in both adults and children (Sardinha et al., 2008; Cooper et al., 2012; Lahjibi et al., 2013; Kim et al., 2018). Insulin is a peptide hormone secreted by the pancreas (specifically, β cells of the islets of Langerhans) that maintains normal blood glucose concentrations throughout the day (glucose homeostasis) (El Sayed and Mukherjee 2017), with insulin resistance typically defined as the state of attenuated biological response to normal or elevated insulin concentrations (Hauner 2002; Wilcox 2005). Insulin resistance is associated with the occurrence of vascular-related conditions including hypertension (Tarray et al., 2014), atherosclerosis (Semenkovich 2006), and endothelial dysfunction (Steinberg et al., 1996), and insulin plays an important role in conduit arteries compliance, modulation of blood flow and relaxation of resistance arterioles (Zheng and Liu 2015). Particularly, insulin is known to stimulate NO production via eNOS activation, and also regulates the release of ET-1 and the proliferation of vascular smooth muscle cells (Janus et al., 2016), which all have an effect on vascular function. Importantly, numerous experimental studies indicate that both acute and short-term inactivity protocols have negative effects on insulin profile. Most studies report significant impairments in insulin action/concentration in response to prolonged sitting (5–9 h) compared with sitting interrupted with regular active breaks (Dunstan et al., 2012; Peddie et al., 2013; Dempsey et al., 2016a; Dempsey et al., 2016b; Henson et al., 2016; Yates et al., 2020), although others have reported no changes (Miyashita et al., 2016). Generally, longer inactivity protocols such as, multiple days of prolonged sitting (Stephens et al., 2011; Larsen et al., 2015), step-reduction (5–14 days) (Krogh-Madsen et al., 2010; Knudsen et al., 2012; Breen et al., 2013; Reynolds et al., 2015; Davies et al., 2018; McGlory et al., 2018), or bed rest (3–9 days) (Stuart et al., 1988; Smorawinski et al., 2000; Alibegovic et al., 2009; Biensø et al., 2012; Dirks et al., 2016) seem to consistently induce signs of insulin resistance compared with observations after one prolonged sitting session. Interestingly, such effects on the insulin profile appear to occur within a similar time frame as inactivity-induced endothelial dysfunction, although very few studies have investigated these outcomes together. As such, Hamburg et al. (2007) showed signs of insulin resistance which were accompanied by vascular impairments following 5 days of bed rest. Contrary to this, Nosova and colleagues observed no significant changes in metabolic biomarkers despite reporting declines in both upper and lower limb vascular function (Nosova et al., 2014).

Considering the number of functions that insulin presents within the vascular endothelium (e.g., NO/ET-1 regulation), it is possible that insulin resistance observed following inactivity protocols might be a mechanism by which insulin resistance mediates inactivity-induced endothelial dysfunction. However, this aspect is not yet clearly elucidated, thus requiring future investigations within the context of acute inactivity, vascular function and insulin concentrations.

### 5.3 Inflammatory markers

Inflammation is strongly associated with vascular dysfunction and risk of CVD (Savoia et al., 2011; Teixeira et al., 2014). In this regard, increases in inflammatory markers such as tumor necrosis factor alpha (TNF-α), interleukin (IL)-1, IL-6, and IL-12 are associated with higher risk of developing hypertension (Jayedi et al., 2019); they also have been shown to have detrimental consequences on vasculature, particularly by promoting atherosclerotic lesions, leukocytes adhesion to the endothelium, and increase in atherosclerotic plaque formation (Galkina and Ley 2009). Furthermore, increases in IL-6 and C-reactive protein (CRP) are known to downregulate NO bioavailability (by attenuating eNOS activity) and upregulate ET-1 levels (Teixeira et al., 2014), and therefore impair endothelial function (Wassmann et al., 2004; Esteve et al., 2007; Hein et al., 2009; Devaraj et al., 2011; Nishiyama et al., 2017). As such, inflammation has been explored and hypothesized as a potential contributing mechanism underlying inactivity-related vascular dysfunction.

Several epidemiological studies showed that sedentary time, both self-reported and objectively measured, is associated with higher levels of several inflammatory biomarkers including leptin, CRP, IL-6, and TNF-α, in both children and adults (healthy and at-risk) (Healy et al., 2011; Allison et al., 2012; Yates et al., 2012; Henson et al., 2013; Falconer et al., 2014; Farah et al., 2016; Gabel et al., 2016). Interestingly, interrupting sedentary time with physical activity breaks, or partially replacing it with standing, or stepping, results in significant reductions in IL-6, CRP, and leptin (Healy et al., 2011; Henson et al., 2018).

Despite the limited number of human randomized controlled trials, some acute and short-term inactivity models also suggest significant modulation of inflammatory markers. Recently, Dogra et al. (2019) reported an increase in salivary IL-8 concentration in response to 4 h of uninterrupted sitting in active young healthy adults. However, no assessments of endothelial function were performed during this sitting intervention, so no direct association with vascular function could be established. Further, using a 2-week step reduction model, short-term inactivity increases circulatory levels of CRP, TNF-α (Breen et al., 2013), and IL-6 concentrations (McGlory et al., 2018) in older adults, but not in young adults (Krogh-Madsen et al., 2010). In agreement with this, short periods of bed rest (i.e., 5 days) appear to not cause detectable changes in systemic inflammatory markers in young healthy adults (Hamburg et al., 2007; Nosova et al., 2014).

Overall, these findings indicate that acute, short and long-term inactivity can induce increases in inflammatory markers particularly in older adults or at-risk populations. Although modulation of inflammatory profile is frequently shown to occur in concomitance with a decline in vascular function in the context of physical inactivity, a causative link between inflammation and inactivity-induced endothelial dysfunction is yet to be established.

### 5.4 Oxidative stress

Oxidative stress has also been suggested as a likely physiological mechanism by which physical inactivity may impair vascular function. Oxidative stress is characterized by an imbalance between elevated reactive oxygen species (ROS) formation and a low antioxidant defence in the body (Pizzino et al., 2017). Elevated levels of ROS are considered a contributing factor for the development of hypertension and atherosclerosis (Cai 2005; Suvorava et al., 2005; Münzel et al., 2017), possibly due to their involvement in vasoconstriction within resistance vessels (Jones and Morice 2000). This idea of oxidative stress as a potential mechanism is supported by observations that supplementation with dietary antioxidants (e.g., vitamin C) prevents declines in endothelial function during prolonged sitting in humans (Thosar et al., 2015a) and attenuates hydrogen peroxide (H_2_O_2_) production and muscle atrophy in rodent models of immobilization (Min et al., 2011). This may be of significance given the known association between low skeletal muscle mass and increased risk of arterial stiffness, coronary artery calcification, and reduced arterial size (Radegran and Saltin 2000; Ko et al., 2016; Yang et al., 2020).

However, there is limited direct evidence for modifications in oxidative stress markers as a consequence of inactivity in humans. Gram et al. (2015) examined the effects of a short-term inactivity intervention (i.e., 2 weeks of unilateral lower limb immobilization) followed by an exercise intervention (i.e., 6 weeks of aerobic cycle training) on mitochondrial ROS production within the vastus lateralis muscle in healthy young and older men. Their findings showed that lower limb immobilization resulted in augmented mitochondrial H_2_O_2_ production, and this was reversed by exercise. In line with this, 5 weeks of bed rest in healthy young men resulted in increased protein carbonylation, a marker of oxidative stress, in vastus lateralis muscle (Dalla Libera et al., 2009). Further, cross-sectional studies reported higher levels of global oxidative stress in sedentary older adults in comparison to non-sedentary (Carmeli et al., 2015). Conversely, a short-term lower limb immobilization protocol showed no significant changes in oxidative stress markers (i.e., protein carbonyls, and 4-hydroxy-2-nonenal adducts) (Glover et al., 2010), and no changes in circulating lipid peroxidation (malondialdehyde) were observed after prolonged sitting in humans (Ballard et al., 2017).

Animal studies report increased production of ROS and enhanced activity of NADPH oxidase (an enzyme responsible for the release of superoxide) in the endothelium and the media of the aortic wall after 6 weeks of reduced physical inactivity, which was accompanied by impaired endothelium-dependent vasodilation and accelerated atherosclerotic plaque development (Laufs et al., 2005). Similarly, hindlimb immobilization (12; 14 days) and unloading (28 days) in rodents also resulted in mitochondrial production of ROS (i.e., hydrogen peroxide) and reduced skeletal muscle mass (Kondo et al., 1991; Lawler et al., 2003; Min et al., 2011). Despite some evidence in animal and human studies that inactivity can affect levels of ROS and NADPH oxidase activity in muscle and vascular tissue, the evidence for the role of oxidative stress on physical inactivity-induced endothelial dysfunction remains to be established.

### 5.5 Haemodynamics and rheological factors

The importance of shear stress and its role in maintaining and regulating vascular health and function is well-established (Gnasso et al., 2001; Thosar et al., 2012; Green et al., 2017). Shear stress is defined as the frictional force that blood flow exerts on the inner layer of the vessel wall (i.e., endothelium) (Fernandes et al., 2018), and is quantified by multiplying shear rate (equal to velocity/diameter) by blood viscosity (Pyke and Tschakovsky 2005). The majority of studies investigating inactivity do not measure blood viscosity, and therefore, shear stress. Typically, shear rate is measured instead, which represents an acceptable surrogate measure of shear stress (Pyke and Tschakovsky 2005). Several studies have shown that exercise-induced increases in shear rate represent an important physiological stimulus for improving endothelial function (Tinken et al., 2009; Tinken et al., 2010; Birk et al., 2012). On the other hand, prolonged sitting has been shown to consistently lead to reductions in shear rate in both upper and lower limb arteries, as described previously in this review. Importantly, continuous/intermittent increases in shear rate, through local heating, prevents declines in endothelial function (measured by FMD) observed during periods of restricted/reduced physical activity (Restaino et al., 2016; Teixeira et al., 2017). This suggests that shear rate is likely underpinning changes in vascular function during periods of inactivity. Indeed, the majority of studies (e.g., Thosar et al., 2015a; Morishima et al., 2016; Morishima et al., 2017) consistently showed that declines in FMD in lower limb arteries are accompanied by reductions in shear rate during prolonged sitting. However, this relationship is less clear in the upper limb arteries, where consistent reductions in shear rate are not always paralleled by impairments in FMD (Thosar et al., 2014; Restaino et al., 2015).

Viscosity is a measure of fluid friction that directly influences the magnitude of shear rate and has also been observed to increase in response to sitting in the lower, but not the upper limb arteries (Hitosugi et al., 2000). This may explain, at least partially, the specificity of the vascular deficits observed in the lower limb arteries during sitting studies. Given that typically lower shear rate results in higher blood viscosity (Chien 1970; Dormandy 1970; Sriram et al., 2014), it is plausible to hypothesize that declines in shear rate observed during sitting may contribute to increases in viscosity, and together these are likely to impact vascular function (e.g., FMD). However, to date, no studies have specifically explored this relationship during inactivity protocols.

In short-term inactivity models, the relationship between blood flow/shear rate and lower limb FMD may be less consistent than in prolonged sitting. For example, Bleeker et al. (2005) did not observe any significant change in blood flow and FMD (corrected for shear rate) in the superficial femoral artery in response to 52 days of bed rest. On the contrary, 12 days of unilateral lower limb immobilization induced increases in both mean shear rate and FMD in the popliteal artery (Rakobowchuk et al., 2011). These contrasting relationships might be driven by the reduction in arterial size observed after longer periods of physical inactivity, which is known to influence mean shear rate and consequently FMD (Pyke and Tschakovsky 2005). However, looking at mean shear rate during inactive periods might not be as informative as looking at its components (anterograde and retrograde shear rate), when trying to establish links with endothelial function. For example, a recent study showed that artificially modulating anterograde and retrograde shear rate patterns alone, without altering mean shear rate, results in increases in FMD (Holder et al., 2019). Indeed, controlled increases and decreases in anterograde shear rate alone result in enhancement and attenuation of FMD, respectively (Tinken et al., 2009). In addition, both acute and prolonged experimental increases in retrograde shear rate reduce brachial FMD/diameter in young healthy men (Thijssen et al., 2009; Thijssen et al., 2015), highlighting the importance of anterograde and retrograde shear rate in modulating vascular function in humans. Indeed, a few studies report declines in anterograde shear rate in brachial and superficial femoral arteries during sitting (Thosar et al., 2014; Thosar et al., 2015a; Thosar et al., 2015b), while others do not (McManus et al., 2015; Carter et al., 2019). However, it is currently difficult to clearly define the impact of altered shear rate patterns (anterograde and retrograde shear rate) in inactivity-induced endothelial dysfunction, as the majority of studies measured only mean shear rate.

The arterial bending of lower limbs that accompany sitting positions can further affect shear rate/stress characteristics. Under normal conditions and based on their geometrical properties, straight conduit arteries (e.g., brachial artery) have typically laminar blood flow (Rabby et al., 2014; Evju and Mardal 2015) and therefore, high wall shear rate/stress (Paszkowiak and Dardik 2003; Chiu and Chien 2011). However, alteration in arterial shape due to arterial bending reduces blood flow and may predispose the artery to experience disturbed blood flow distal to the bending site (Walsh et al., 2017). Disturbed blood flow, a nonuniform and irregular flow, is mainly characterized by low and reciprocating shear stress (due to a periodic flow in which its velocity oscillates in backward and forward direction) (Chiu and Chien 2011), and is typically observed in areas prone to atherosclerosis (i.e., arterial branches and curvatures), such as femoral arteries (Chiu and Chien 2011; Heo et al., 2014). Accumulating evidence indicates that disturbed blood flow has negative effects on the vasculature (Chiu and Chien 2011; Heo et al., 2014), including reductions of eNOS expression (Gambillara et al., 2006), increase of ROS production (Heo et al., 2011), and promotion of endothelial cell activation and apoptosis (Jenkins et al., 2013).

Interestingly, sitting studies report reductions in blood flow and/or shear rate once participants transition from supine to the sitting position (Restaino et al., 2016; Vranish et al., 2017; Vranish et al., 2018), and a recovery of blood flow when moving back to supine position (Morishima et al., 2017). In agreement with this, Walsh et al. (2017) have shown that when the leg is bent at 90° at the hip and knee during the lateral lying down position, there is a significant decline in popliteal artery FMD, similarly to what has been observed during sitting. This was further accompanied by reductions in mean, anterograde, and retrograde shear rate within the first hour of arterial bending, which partially reflects what has been observed during prolonged sitting (Thosar et al., 2014; Thosar et al., 2015a; Thosar et al., 2015b; Morishima et al., 2020a; Morishima et al., 2020b). This highlights that turbulent flow patterns induced by arterial bending might also contribute to declines in endothelial function during prolonged sitting.

In summary, there is some evidence to suggest that reduced anterograde shear rate, higher blood viscosity, and disturbed blood flow may contribute to endothelial dysfunction during physical inactivity periods, particularly during prolonged sitting, but more studies are needed to establish these relationships.

### 5.6 Hydrostatic pressure

As outlined above, the vasculature in the upper and lower limb arteries seems to be differentially affected during periods of inactivity (Thosar et al., 2014; Restaino et al., 2015; Climie et al., 2018). The mechanisms underlying these differences are not clear, however, it is known that blood pressure in the lower limbs is generally higher in comparison with upper limbs (Sheppard et al., 2019) independently of the body posture adopted (Pollack and Wood 1949; Gemignani et al., 2012). These differences in blood pressure, particularly during sitting and standing (Pollack and Wood 1949; Gemignani et al., 2012), may be attributed to elevated hydrostatic pressure, which is a component of blood pressure that refers to the force that blood exerts on the blood vessels due to the effect of gravity (Badeer 2001; Darwish and Lui 2020). Indeed, *in vitro* studies show that whilst physiological hydrostatic pressure (i.e., ∼11 mmHg) for short periods (i.e., 45 min) protects endothelial cells integrity (Muller-Marschhausen et al., 2008), sustained exposure stimulates the proliferation of human endothelial cells (Acevedo et al., 1993; Schwartz et al., 1999)—a response that has been linked to atherosclerosis (Fuster et al., 2010). Furthermore, elevated hydrostatic pressure (i.e., ≥50 mmHg) results in a reduction in endothelial barrier function (Prystopiuk et al., 2018) and VE-cadherin protein expression (Ohashi et al., 2007), both of which have been associated with endothelial cells apoptosis (Carmeliet et al., 1999). A study in humans reported that acute exposure of the upper limb arteries to increased hydrostatic pressure induced declines in FMD (Padilla et al., 2009), suggesting a link between hydrostatic pressure and endothelial dysfunction.

The lower body vasculature is chronically exposed to higher hydrostatic pressure and this may explain why lower limb arteries have reduced vascular responsiveness and increased vascular susceptibility to inactivity-induced detrimental effects (Bleeker et al., 2005; Thosar et al., 2014; Restaino et al., 2015; Climie et al., 2018). However, it is currently not clear to what extent hydrostatic pressure contributes to the difference in vascular function responses between upper and lower limbs in the context of physical inactivity. In addition, it has been suggested that increased hydrostatic pressure in lower extremities during sitting may further induce blood pooling (Padilla and Fadel 2017), which refers to the slowing of blood within the venous circulation due to lack of skeletal muscle activity (Tansey et al., 2019). The increase in hydrostatic pressure within the vascular tree forces the fluid to filter out from blood vessels into the interstitial space, leading to localized edema (Hall and Hall 2020). In support of this, several studies have shown that sitting-induced declines in FMD are accompanied by blood pooling in lower limbs, as reflected by marked increase in calf (Restaino et al., 2015; Vranish et al., 2017; Kruse et al., 2018; Credeur et al., 2019; O’Brien et al., 2019) and ankle circumference (Morishima et al., 2016).

Increased lower limb blood pooling contributes to a cascade of physiological events, in particular a reduction in central venous return, ventricular filling, stroke volume and blood pressure, which results in an increase in sympathetic nervous system activity by stimulation of baroreflexes (Freeman 2006; Stone et al., 2016). This can be clinically relevant given that increase in sympathetic nervous system activity, which is known to induce peripheral vasoconstriction, affects vascular function through possible inhibition of shear-mediated NO production (Amiya et al., 2014).

Overall, sitting may induce increase in hydrostatic pressure in lower limb blood vessels that results in leg blood pooling. Both hydrostatic pressure and blood pooling appear to have negative effects on the vasculature, and therefore, they might contribute to the underlying physiological mechanisms by which sitting affects lower limb vascular function.

## 6 Long-term consequences of physical inactivity

### 6.1 Arterial remodelling and hypertension

Arterial remodelling refers to a set of functional and structural adaptations of the blood vessel wall in response to disease, injury, aging (Van Varik et al., 2012) and also exposure to regular periods of physical activity/inactivity (Thijssen et al., 2011; Thijssen et al., 2012). Vascular remodelling includes inward remodelling (reduction in arterial lumen) and outward remodelling (increase in arterial lumen), and depending on variations in vascular wall thickness, vessels may undergo hypotrophic (reduction in arterial wall), eutrophic (no alteration in arterial wall), and hypertrophic (increase in arterial wall) remodelling (Van Varik et al., 2012).

Vascular inward eutrophic remodelling in blood vessels appears to take place in response to short-term inactivity protocols. In particular, bed rest, limb immobilization and step reduction result in declines in arterial diameter in the brachial, femoral (common and superficial), and popliteal arteries (Sugawara et al., 2004; Bleeker et al., 2005; Hamburg et al., 2007; Rakobowchuk et al., 2011; Boyle et al., 2013), without alterations in femoral artery intima-media thickness and intima-media thickness/lumen ratio (Sugawara et al., 2004). Importantly, there is currently well-established evidence linking vascular inward remodelling to hypertension (Baumbach and Heistad 1989; Skov et al., 1992; Rizzoni et al., 1996; Mulvany 1999; Intengan and Schiffrin 2001; Rizzoni et al., 2009; Schiffrin 2012; Brown et al., 2018). However, the literature focuses particularly on the link between hypertension and inward remodelling of small arteries, such as resistance and cerebral arteries, while the link between hypertension and inward remodelling of conduit arteries (e.g., femoral, and brachial arteries) is not yet established. Nonetheless, inactivity might contribute to the development of hypertension through the inward remodelling process of resistance vessels. In fact, individuals with essential hypertension show signs of inward eutrophic remodelling in resistance vessels, which result from an increased media-to-lumen ratio without altering the cross-sectional area of the tunica media (Mulvany 1999; Brown et al., 2018). This type of alteration seems to occur in both the upper and lower body vasculature in response to inactivity protocols (i.e., bed rest, and limb immobilization), as indicated by local reduction in peak blood flow (Hamburg et al., 2007; Birk et al., 2013), which is a surrogate measure of resistance vessel remodelling (Thijssen et al., 2011). This may be clinically relevant considering that structural alteration of resistance arteries is a powerful predictor of cardiovascular events in high-risk populations (Rizzoni et al., 2003; Mathiassen et al., 2007). Indeed, several epidemiological studies show an association between sedentary behaviour and increased risk of developing hypertension (Beunza et al., 2007; Lee and Wong 2015; Lim et al., 2017).

Collectively, these observations highlight that inactivity can induce vascular remodelling in resistance and conduit arteries and this is likely linked to increased incidence of hypertension in sedentary individuals.

### 6.2 Atherosclerosis

Atherosclerosis is a chronic inflammatory disease characterized by a progressive accumulation of fatty deposits and fibrous components (forming plaques) in the inner layer of the artery (tunica intima), resulting in narrowing of the lumen and thickening of the arterial wall (Ross 1999; Lusis 2000; Rafieian-Kopaei et al., 2014). This results in inadequate blood perfusion to organs (e.g., heart, brain) or extremities (e.g., lower limbs) (Ross 1999; Rafieian-Kopaei et al., 2014) that can lead to serious complications such as heart attack, stroke, or PAD (Banerjee and Chimowitz 2017; Shu and Santulli 2018; Palasubramaniam et al., 2019). In particular, PAD is characterized by the accumulation of atherosclerotic plaques in the peripheral arteries in the lower extremities (Zemaitis et al., 2017). Several studies have shown that atherosclerotic lesions typically develop in areas characterized with low anterograde shear stress (Gnasso et al., 1997; Cheng et al., 2006; Heo et al., 2014) and high OSI (Wu et al., 2004). These atherosclerosis-prone areas are typically vessel curvatures, bifurcations, and branch points in which the blood flow changes its pattern from laminar to disturbed blood flow (Owen and Roberts 2007; Jenkins et al., 2013). Alterations in blood flow patterns predispose the vasculature to accumulation of atherosclerotic plaques by inducing expression of atherogenic genes and proteins (Nigro et al., 2011). Indeed, a recent longitudinal study has shown a higher prevalence of atherosclerosis within the femoral arteries in comparison with coronary and carotid arteries, and a more robust association between femoral atherosclerosis and cardiovascular risk factors (Laclaustra et al., 2016).

During sitting, the bending of the lower limbs further predisposes those arteries (e.g., popliteal and femoral arteries) to atherogenic disturbed blood flow distal to the bending site (Walsh et al., 2017), which contributes to altered shear patterns and reduced endothelial function (Chiu and Chien 2011; Jenkins et al., 2013). In line with this and as described previously in this review, acute and short-term inactivity interventions consistently result in decreases in shear rate and impairments in vascular function in the lower limb arteries (e.g., Thosar et al., 2014; Thosar et al., 2015a; Thosar et al., 2015b; Restaino et al., 2015; Morishima et al., 2016). This is also relevant given that impairments in endothelial function in the popliteal artery seem closely related to increases in intima-media thickness (Iwamoto et al., 2016), which is a valuable marker of subclinical atherosclerosis (de Groot et al., 2004; Harrington et al., 2010; Bauer et al., 2012). Collectively, these findings suggest that lower limb atherosclerosis is likely one of the long-term consequences of inactivity-induced endothelial dysfunction.

In agreement with this, a large population-based study showed a dose-response relationship between sedentary time and increased risk of atherosclerotic PAD (Unkart et al., 2020). Further studies in both healthy and at-risk populations have reported that time spent in sedentary activities (e.g., TV viewing) is directly associated with the progression of coronary artery calcification, and carotid intima-media thickness (Kozàkovà et al., 2010; Delaney et al., 2013; García-Hermoso et al., 2015; Diaz et al., 2016; Lazaros et al., 2019). Other studies have shown associations between sedentary time and either low ankle-brachial index values or PWV, which are both non-invasive tools for detecting PAD development, and arterial stiffness, respectively (Horta et al., 2015; Kulinski et al., 2015; Ahmadi-Abhari et al., 2017). The impact of physical inactivity on the progression of PAD has also been demonstrated in epidemiological and experimental studies in spinal cord injury patients (Bell et al., 2011; Su et al., 2015).

Collectively, these findings highlight that vascular alterations induced by inactivity may play an important role in the development of atherosclerosis, particularly in the lower limbs.

### 6.3 Skeletal muscle function/mass

It is well-established that increased sedentary behaviour is associated with reduced skeletal muscle mass and greater risk of developing sarcopenia (Gianoudis et al., 2015; Aggio et al., 2016; Smith et al., 2020). Sarcopenia is an age-related condition characterized by a progressive reduction of skeletal muscle mass and function (Santilli et al., 2014; Cruz-Jentoft and Sayer 2019) that can develop as early as the 4^th^ decade of life (Metter et al., 1997). Studies have shown that inactivity interventions such as bed rest (10 days–17 weeks) (LeBlanc et al., 1992; Paddon-Jones et al., 2004; Kortebein et al., 2008; Coker et al., 2015), step reduction (14 days) (Krogh-Madsen et al., 2010; Breen et al., 2013; Davies et al., 2018; McGlory et al., 2018), unilateral arm (Kitahara et al., 2003; Birk et al., 2013) and leg immobilization (5–21 days) (Glover et al., 2008; Suetta et al., 2009; Dirks et al., 2014), result in reductions in muscle protein synthesis rate, muscle function, and muscle mass, which occur mainly in the lower limbs.

Reduced blood perfusion during inactivity, which may be the consequence of multiple factors including impairments in NO signalling and skeletal muscle capillarity (Gram et al., 2014), may contribute to the muscular functional impairments observed, particularly within the context of sarcopenia (Zempo et al., 2017; Hendrickse and Degens 2019). Consistent with this, Rasmussen et al. (2006) showed increases in leg blood flow and muscle protein synthesis in response to insulin infusion in young healthy adults and further reported that insulin-induced increases in blood flow were positively correlated with changes in muscle protein synthesis. Similarly, Volpi et al. (2000) showed that an increase in endogenous insulin concentrations due to amino acid-glucose administration resulted in increased blood flow and muscle protein anabolism in young individuals. Importantly, when insulin or amino acids were infused in combination with the NO donor sodium nitroprusside, improvements in muscle protein anabolism in both young and elderly adults were observed (Timmerman et al., 2010; Dillon et al., 2011). Collectively, these findings support a role for blood flow in regulating muscle protein anabolism. Therefore, the decline in blood flow that results from physical inactivity and particularly sitting may contribute to impairments in protein anabolism and loss of muscle mass linked to sedentary behaviour. Another aspect that may contribute to functional and structural impairments in skeletal muscle tissue is arterial stiffness. Several studies have indeed shown negative associations between arterial stiffness and skeletal muscle mass/strength (Ochi et al., 2010; Im et al., 2017; Zhang et al., 2019). As such, given the experimental evidence showing increases in arterial stiffness (i.e., PWV) in response to short bouts of sitting (Credeur et al., 2019; Evans et al., 2019), this may indicate another mechanism by which inactivity can contribute to muscular impairments. However, the links between vascular and muscular dysfunction in the context of inactivity have not been experimentally established. Currently, it is not clear whether inactivity-induced vascular dysfunction is causally contributing to a decline of functional and structural aspects of skeletal muscle tissue.

## 7 Preventive strategies for counteracting negative effects of physical inactivity

### 7.1 Physical exercise

Physical activity is naturally one of the main interventions used to attempt to prevent vascular detrimental effects of prolonged inactivity. Many epidemiological studies have highlighted the beneficial associations between physical activity and prognostic outcomes for vascular health, whilst sedentary activity tends to be associated with poorer outcomes (Gomez-Marcos et al., 2014; Horta et al., 2015). A longitudinal study in elderly adults, reported that increases in arterial stiffness over a 5-year period (specifically, 0.76 m⋅s^−1^ increase in carotid-femoral PWV) were significantly attenuated by 0.02 m⋅s^−1^ for every additional hour per week of moderate-to-vigorous physical activity, whilst increased sedentary time was associated with higher rates of arterial stiffness (Ahmadi-Abhari et al., 2017). Furthermore, among highly active individuals the association between sedentary time and the relative risk for all-cause mortality is 30% lower in comparison with less active individuals (Biswas et al., 2015). Moreover, replacing 1 h per day of sitting with moderate-to-vigorous cycling exercise resulted in improvements in biomarkers of endothelial dysfunction (i.e., ICAM-1, E-selectin) in young healthy and at-risk adults (Duvivier et al., 2018). Similarly, in children, replacing 1 h per day of sedentary time with light/moderate-to-vigorous physical activity showed beneficial effects on the clustering of cardio-metabolic risk (i.e., systolic and diastolic blood pressure, waist circumference, and HDL cholesterol) (Wijndaele et al., 2019). Overall, these studies indicate that habitual physical activity even in small increments can have beneficial long-term consequences for cardiovascular health (Palombo and Kozakova 2016).

**FIGURE 3:**
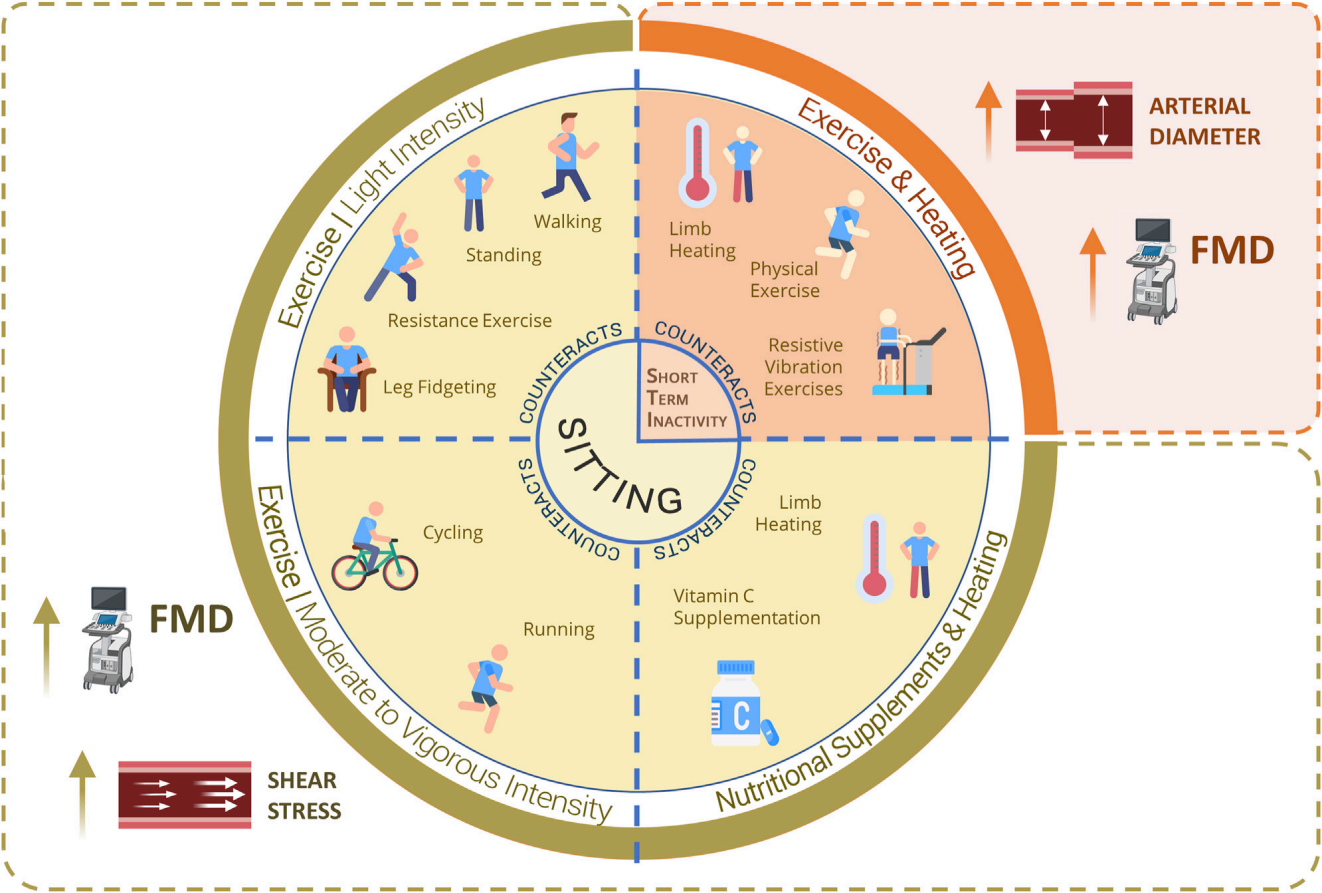
Summary of intervention strategies that have been shown to be effective at preventing detrimental effects on vascular health during periods of physical inactivity. Standing, intermittent leg fidgeting, walking, running, cycling, resistance exercises, limb heating and vitamin C supplementation were shown to be effective at improving FMD and/or shear stress/rate in the context of prolonged sitting. Resistive vibration exercises, limb heating and general physical activity were shown to be effective at preventing declines in FMD and/or arterial diameter during inactivity protocols such as bed rest, step-reduction, and leg immobilization. *FMD: Flow-mediated dilation*.

Human randomized experimental studies have further assessed the efficacy of bouts of physical activity as a strategy to interrupt prolonged sitting (Figure 3) (Table 3). Specifically, interrupting sitting every 30–60 min with short walking/cycling breaks (2–10 min) has been shown to be effective in preventing declines in lower limb endothelial function across different populations (Thosar et al., 2015a; McManus et al., 2015; Hartman et al., 2021). Similarly, 3 min of simple resistance activities, such as half squats, calf raises, and single knee raises, every 30 min (within a 7-h sitting period) are sufficient to prevent sitting-induced declines in femoral FMD, resting blood flow and shear rate in middle-aged/elderly adults (Climie et al., 2018), as well as reported benefits in young women with PCOS (Taylor et al., 2020) and adults with type 2 diabetes (Taylor et al., 2021). Furthermore, intermittent leg fidgeting (1 min on, 4 min off during a 3-h sitting protocol) has been shown to ameliorate popliteal FMD and attenuate declines in shear rate in young healthy adults (Morishima et al., 2016). The beneficial impact of physical activity is also observed in upper limb arteries, albeit to a lesser extent, with a recent study reporting that callisthenic exercises (2-min set every 20 min) during 1.5 h of sitting elicited an increase in brachial artery shear rate in young healthy adults (Carter et al., 2017). Finally, a more recent study has found, within the same population, that performing stair climbing breaks (5 min every hour) during a 4-h sitting intervention, improves FMD and shear rate in brachial and popliteal artery, respectively (Cho et al., 2020). However, not all studies have shown such positive effects. For example, Kruse and colleagues found no benefits in the popliteal artery with brief intermittent bouts of light-intensity desk pedalling during a 4-h sitting period in early middle-aged overweight/obese adults (Kruse et al., 2018), and Carter and colleagues did not observe any significant positive effects on a range of vascular outcomes (e.g., FMD and shear rate), with the exception of marginal benefits on blood flow and vascular conductance, in young adults when utilising activity breaks (i.e., 2-min walking every 30 min; 8-min walking every 120 min) during their 4-h prolonged sitting protocol (Carter et al., 2019). Collectively, these findings may indicate that higher exercise intensity/duration may be needed in some populations in order to counteract the negative vascular effects induced by uninterrupted prolonged sitting.

**TABLE 3:** The effects of counteracting interventions on inactivity-induced vascular dysfunction.

Outcome measures							
References	Population (N)	Artery	Inactivity model	Intervention	%FMD	Shear rate/stress	Other
Ballard et al. (2017)	Young healthy (11)	SFA	SIT (3 h)	Running (45 min, prior sitting)	↑	↑	ET-1: ↔
Bleeker et al. (2005)	Young healthy (16)	SFA - CFA - BA- CCA	Bed rest (52 days)	Resistive vibration exercise (twice daily for 30 min)	SFA: ↔		Diameter (SFA: ↑; CFA: ↑; BA: ↔; CCA: ↔)
Caldwell et al. (2020)	Young healthy (26)	SFA	SIT (2 h)	Standing (replacing sitting with standing); Foot heating (during sitting at 49°C)	Standing: ↑; Foot heating: ↑	Standing: ↑; Foot heating: ↑	ET-1 (Standing: ↔; Foot heating: ↔); PWV (Standing: ↑; Foot heating: ↔)
Carter et al. (2017)	Young healthy (10)	BA	SIT (∼1.5 h)	Callisthenics exercises (2 min every 20 min)	↔	↑	
Carter et al. (2019)	Young healthy (15)	SFA	SIT (4 h)	2WALK (2-min walking break every 30 min); 8WALK (8-min walking break every 2 h)	2WALK: ↔; 8WALK: ↔	2WALK: ↔; 8WALK: ↔	
Cho et al. (2020)	Young healthy (12)	PA - BA	SIT (4 h)	Stair climbing (5 min every h)	BA: ↑	PA: ↑	
Climie et al. (2018)	Late middle-aged at risk (19)	SFA - BA	SIT (5 h)	SRA (3 min every 30 min)	SFA: ↑; BA: ↔	SFA: ↔; BA: ↔	ET-1: ↓
Cohen et al. (2021)	Young healthy (31)	SFA	Leg immobilization (14 days)	BFR (twice daily for 30 min); BFR + EMS (twice daily for 30 min)	↔	↔	Diameter: ↔
Decker et al. (2021)	Young healthy (26)	CFA	SIT (1.5 h)	Vitamin C supplementation (1 g, 90 min prior to the testing session)			PLM-induced hyperaemia [LBF_Δpeak_ (men: ↑; women: ↔)]
Evans et al. (2019)	Young healthy (20)		SIT (3 h)	Calf exercise (10 calf raises every 10 min)			PWV: ↔; TSI: ↑
Garten et al. (2019a)	Young healthy (10)	CFA	SIT (3 h)	Cycling (∼45 min, prior sitting)			PLM-induced hyperaemia (∆LBF: ↑; LBF AUC: ↑)
Hartman et al. (2021)	Elderly at risk (24)	SFA		16-week intervention (reduced sitting + physical activity)	↑		Diameter: ↑
Hartman et al. (2021)	Elderly at risk (24)	SFA	SIT (3 h)	Walking (2 min every 30 min)	↑		
Hyldahl et al. (2021)	Young healthy (21)	CFA	Leg immobilization (10 days)	Thigh heating (2 h, daily)			Diameter: ↑; PLM hyperaemia: ↑; VEGF: ↑; ET-1: ↔
Kowalsky et al. (2019)	Late middle-aged at risk (14)		SIT (4 h)	SRA (every h)			PWV: ↔
Kruse et al. (2018)	Early middle-aged (overweight/obese) (13)	PA	SIT (4 h)	Standing (10 min every h); desk pedalling (10 min every h)	Standing: ↔; desk pedalling: ↔	Standing: ↔; desk pedalling: ↔	
McManus et al. (2015)	Young healthy girls (9)	SFA	SIT (3 h)	Cycling (10 min every h)	↑	↔	
Morishima et al. (2017)	Young healthy (15)	PA	SIT (3 h)	Cycling (45 min, prior sitting); standing (replacing sitting with standing)	Cycling: ↑; standing: ↑	Cycling: ↑; standing: ↑	
Morishima et al. (2016)	Young healthy (11)	PA	SIT (3 h)	Intermittent leg fidgeting (cycle: 1 min on, 4 min off)	↑	↑	
Morishima et al. (2020a)	Young healthy (19)	PA	SIT (3 h)	Fish oil supplementation (8 capsules per day, for 8 weeks)	↔	↔	
Peddie et al. (2021)	Young healthy (18)	PA	SIT (6 h)	Standing (replacing sitting with standing); activity breaks (2 min of walking every 30 min)	Standing: ↔; activity breaks: ↔	Standing: ↑; activity breaks: ↑	Blood flow: (Standing: ↑; activity breaks: ↑)
Restaino et al. (2015)	Young healthy (11)	PA - BA	SIT (6 h)	Walking (10 min, after sitting)	PA: ↑; BA: ↔	PA: ↑; BA: ↔	
Restaino et al. (2016)	Young healthy (10)	PA	SIT (3 h)	Foot heating (during sitting at 42°C)	↑	↑	
Taylor et al. (2020)	Young at risk (13)	SFA	SIT (3.5 h)	SRA (3 min every 30 min)	↔	↑	
Taylor et al. (2021)	Middle-aged/elderly at risk (24)	SFA	SIT (7 h)	SRA3 (3 min of SRA every 30 min); SRA6 (6 min of SRA every 60 min)	SRA3: ↑; SRA6: ↔	SRA3: ↑; SRA6: ↑	ET-1 (SRA3: ↔; SRA6: ↔)
Teixeira et al. (2017)	Young healthy (13)	PA	Step reduction (5 days)	Intermittent foot heating (3 times [30 min each] per day at 42°C)	↑		Diameter: ↔
Thosar et al. (2015a)	Young healthy (12)	SFA	SIT (3 h)	Walking (5 min every h)	↑	↔	
Thosar et al. (2015b)	Young healthy (11)	SFA	SIT (3 h)	Vitamin C supplementation (1 tablet of 1 g after 30 min; 1 tablet of 500 mg after 90 min)	↑	↔	
Tremblay et al. (2019)	Young Healthy (11)	SFA	SIT (0.5 h)	Replacing normal sitting with sitting cross-legged	↔	↑	PWV: ↔

BA, brachial artery; BFR, blood flow restriction; CCA, common carotid artery; CFA, common femoral artery; EMS, electrical muscle stimulation; ET-1, endothelin-1; LBF, leg blood flow; PA, popliteal artery; PLM, passive leg movement; PWV, pulse wave velocity; SFA, superficial femoral artery; SIT, sitting; SRA, simple resistance activities; TSI, tissue saturation index; VEGF, vascular endothelial growth factor.

The performance of physical activity before or immediately after a prolonged period of sedentary behaviour also seems to be beneficial for vascular function. For example, Restaino et al. (2015) reported that 10 min of walking at the end of a 6-h sitting trial reversed the decline in mean blood flow (resting, and hyperaemic), shear rate, and popliteal artery FMD, with no benefits detected in the brachial artery (Restaino et al., 2015). Recent studies also showed that 45 min of aerobic exercise on a cycle ergometer (Morishima et al., 2017) and on a treadmill (Ballard et al., 2017) prior to a 3-h sitting bout effectively prevented reductions in FMD on the popliteal and superficial femoral artery, respectively. Importantly, exercise-induced vascular improvements during prolonged sitting are also accompanied by metabolic changes, such as reductions in postprandial plasma glucose (Bailey and Locke 2015; Bhammar et al., 2017; Champion et al., 2018) and insulin concentrations (Dunstan et al., 2012; Peddie et al., 2013; Larsen et al., 2015; Henson et al., 2016; Yates et al., 2020), as well as benefits to systolic/diastolic BP (Larsen et al., 2014; Zeigler et al., 2015; Dempsey et al., 2016a; Zeigler et al., 2016; Bhammar et al., 2017; Champion et al., 2018) and vascular biomarkers (i.e., ET-1) (Climie et al., 2018). Overall, this highlights a crucial role that physical activity can play in preventing peripheral vascular and cardiometabolic damages induced by prolonged sitting.

In the context of longer periods of physical inactivity, there are only a few studies investigating the efficacy of controlled physical activity interventions. Reynolds et al. (2015) observed that 1 day of normal ambulatory activity (>12,000 steps per day) following 5 days of step reduction (<4,000 steps per day) was not sufficient to exert any significant improvement in insulin sensitivity (which was reduced by inactivity), or insulin-stimulated blood flow responses and vascular conductance in the femoral and brachial artery (Reynolds et al., 2015). As such, longer periods of inactivity may require more intense physical activity or a greater duration of time (i.e., number of days) to recover from the inactivity-induced vascular impairments, but more studies are needed to establish this. Alternatively, exercising with the use of a resistive vibration footplate, specifically by performing squats, heel raises, toe raises, and ‘kicks’ (twice daily for 30 min), during horizontal bed rest (52 days), is reported to prevent arterial size reduction in the lower limbs (Bleeker et al., 2005). A similar physical intervention has been used during 60 days of head-down tilt bed rest and resulted in attenuations in the decline of the superficial femoral artery diameter and prevented the increase in intima-media thickness (in carotid, and superficial femoral artery). However, when the exercise was performed without the use of the resistive vibration footplate, it was not sufficient to attenuate the inactivity-induced reduction in diameter of the superficial femoral artery (van Duijnhoven et al., 2010a; van Duijnhoven et al., 2010b). Similarly, a combination of aerobic (35 min running; 3–4 times per week) and resistance exercise (∼30 min; every 3 days) prevented increases in circulating endothelial cells, a biomarker of endothelial damage, and declines of endothelium-dependent vasodilation in cutaneous microcirculation during head-down tilt bed rest (Demiot et al., 2007). Finally, a recent study has shown that a 16-week intervention of reduced sitting combined with low-intensity physical activity improved the FMD response and increased the diameter of the superficial femoral artery in elderly individuals with increased cardiovascular risk (Hartman et al., 2021).

In addition to the benefits described for the peripheral vasculature, there is also some evidence showing physical activity-induced improvements in cerebrovascular function during prolonged sitting. For example, Carter et al. (2018) reported beneficial effects on brain vasculature in response to walking breaks (i.e., 2-min walking breaks every 30 min, and 8-min walking breaks every 120 min). Interestingly, shorter and more frequent walking breaks improved cerebral autoregulation and prevented the reductions in MCAv observed following 4 h of uninterrupted sitting. On the other hand, longer and less-frequent walking breaks were not sufficient to prevent sitting-induced declines in MCAv. This suggests that frequency of physical activity may be important to prevent declines in blood flow during prolonged sitting (Carter et al., 2018). Additionally, performing moderate-intensity walking (30 min) alone or in combination with walking breaks during prolonged sitting (8 h) has been shown to ameliorate changes in MCAv towards the end of the sitting period in comparison to sitting alone (Wheeler et al., 2019).

Collectively, there is encouraging evidence suggesting that different types of physical activity are effective in preventing impairments in both peripheral and cerebral vasculature function in the context of acute and short-term inactivity periods in young healthy adults, but benefits in other populations need further research.

### 7.2 Standing

Office-based workers spend a considerable amount of time seated daily (Fisher et al., 2018) and sit-stand workstations have proven effective in reducing sitting time in the office environment (Hedge and Ray 2004; Alkhajah et al., 2012; Pronk et al., 2012), although their efficacy at reducing vascular impairments is less clear. For example, Morishima et al. (2017) investigated the effects of two different 3-h interventions (standing vs. sitting) on lower limb vascular function in young healthy individuals. Interestingly, prolonged standing was effective in preventing sitting-induced declines in shear rate and FMD in the popliteal artery (Morishima et al., 2017). Similarly, two more recent studies showed that replacing sitting with standing for 2–6 h was effective in preventing the sitting-induced declines in vascular function in lower limb arteries (Caldwell et al., 2020; Peddie et al., 2021). Other studies have further shown that alternating sitting and standing (every 30/60 min) is an effective strategy for reducing SBP (Zeigler et al., 2016), DBP, MAP, and carotid-ankle PWV (Barone Gibbs et al., 2017). However, Kruse and colleagues reported that interrupting 4 h of sitting with standing (10-min bouts of standing at the beginning of each hour) was not effective at preventing sitting-induced decline in FMD in the popliteal artery in sedentary middle-aged adults (Kruse et al., 2018). This suggests that populations that are chronically sedentary may need longer and/or more intense physical interventions (rather than standing) to prevent transient vascular impairments caused by sitting. Graves et al. (2015) showed that the use of a sit-stand workstation during 8 weeks in a group of healthy middle-aged adults resulted in only a significant reduction in total cholesterol, whilst BP and brachial artery FMD were not affected significantly. However, no objective measurements of sitting, standing, and walking time were taken in that study.

Despite some of the experimental studies indicating vascular benefits of standing, epidemiological studies suggest that long periods in this particular posture may be deleterious for vascular health. Indeed, the majority of studies report associations between prolonged standing at work and high prevalence of venous vascular diseases (Krijnen et al., 1997; Tomei et al., 1999; Tüchsen et al., 2000; Tüchsen et al., 2005; Yun et al., 2018). Furthermore, standing at work has been associated with increase in carotid intima-media thickness (Krause et al., 2000), and decline in blood pressure (measured on the wrist), which is suggested to be the result of impaired venoconstriction and blood pooling in the lower extremities (Ngomo et al., 2008).

Overall, standing may be a valid alternative for preventing vascular dysfunction associated with short periods of prolonged sitting. However, recommendations of standing desks in the office environment, as a way to break sitting time, need to be accompanied by more specific guidelines on frequency and duration of standing that elicit maximal benefits and minimize other vascular complications.

### 7.3 Thermal stress

Local heating has been shown to result in rapid increases in blood flow, shear rate and FMD in upper and lower limb conduit arteries (Tinken et al., 2009), similarly to those occurring as a result of cycling and handgrip exercise (Tinken et al., 2009). In the context of prolonged sitting, unilateral foot heating exposure (at 42–49°C) during an uninterrupted sitting protocol (2–3 h) prevented the sitting-induced reduction in mean shear rate and FMD in the popliteal and femoral artery in a group of healthy young adults (Restaino et al., 2016; Caldwell et al., 2020). In line with this, intermittent foot heating (at 42°C; 3 times per day) during 5 days of reduced activity (from >12,000 to <4,000 steps per day) appears effective at preventing inactivity-induced decline in the popliteal artery FMD, although no significant changes in reactive hyperaemia and arterial diameter were detected (Teixeira et al., 2017). The positive effects observed here are likely due to the increase in leg blood flow and mean shear rate in response to each heating session (Teixeira et al., 2017). Finally, a daily 2-h heating treatment (via pulsed shortwave diathermy on the vastus lateralis) during 10 days of leg immobilization increases the hyperaemic response to passive leg movement, as well as attenuates the reductions in femoral artery diameter and VEGF (Hyldahl et al., 2021).

Collectively, these findings indicate that local heating interventions may also be an effective strategy to prevent endothelial dysfunction induced by acute and short-term physical inactivity, by modulating blood flow and shear rate. However, from a practical perspective, it might be a challenging alternative and not feasible in most situations (e.g., office workplace).

### 7.4 Diet

Eating and sitting are often concurrent behaviours, presenting a unique opportunity to use dietary strategies to counteract the negative effects of sitting. Despite this, the topic has received little attention to date. Of the limited studies that have explored this in human participants, Thosar and colleagues showed that vitamin C ingestion during 3 h of uninterrupted sitting (1 tablet of 1 g after 30 min; 1 tablet of 500 mg after 90 min) was effective in preventing sitting-induced reductions in shear rate (i.e., anterograde and mean) and FMD in the superficial femoral artery in healthy young men (Thosar et al., 2015a). Given the high antioxidant capacity of vitamin C (Kim and Lee 2004), their study highlighted that sitting-induced impairments in endothelial function may be at least partially driven by increased oxidative stress within the vasculature, although this was not directly investigated within this study. Interestingly, a more recent study showed that vitamin C ingestion (1 g) 90 min prior to the testing session was effective in preventing sitting-induced reductions in leg microvascular function. However, these positive effects were observed only in male participants and not in female participants (Decker et al., 2021).

In addition, Morishima and colleagues investigated the effects of 8 weeks of fish oil supplementation (containing omega-3 polyunsaturated fatty acids) on popliteal artery vascular function following a 3-h sitting protocol (Morishima et al., 2020a). They reported that fish oil was ineffective in counteracting sitting-induced endothelial dysfunction, with no improvements in shear rate or FMD observed (Morishima et al., 2020a). Considering the beneficial properties of omega-3 fatty acid supplementation on vascular function (Wang et al., 2012), even for a shorter period of time (i.e., 3 weeks) (Żebrowska et al., 2015), the findings from the study of Morishima et al. (2020a) may suggest that the vasculature (especially in lower limb arteries) responds to short-term dietary interventions differently within the context of inactivity. However, caution must be taken before drawing these conclusions as the evidence available is currently very scarce.

## 8 Summary: Conclusion

Evidence from epidemiological and observational studies show that sedentary behaviour is a major modifiable risk factor for future chronic diseases, increasing the risk of CVD and all-cause mortality, independently of physical activity. Sedentary behaviour is alarmingly high, particularly in older adults (accounting of more than 720 million people worldwide) (Kamiya et al., 2020), with almost 70% estimated to spend more than 8 h daily on sitting or low energy expenditure activities (Harvey et al., 2013). This situation is expected to worsen as demographic projections show in the near future a considerable increase in the elderly population worldwide (Kamiya et al., 2020). Importantly, the performing of physical exercise, particularly at low volume and intensity, may not be sufficient to prevent the harmful impact that sedentary behaviour has on health. However, there is evidence indicating that physical exercise at higher volume and intensity may instead be effective in attenuating but not always eliminating the increased mortality risks associated with sedentary time. Therefore, considering the detrimental health impact of sedentary behaviour, reducing sedentary time in daily life is a crucial first step for improving health status and quality of life. Currently, our understanding of the physiological abnormalities that underlie sedentary behaviour and direct links to long-term health issues is only starting to emerge. This combined knowledge will be critical for the development of effective strategies to offset the negative impact of sedentariness across the lifespan.

Acute (hours) and short-term (days; weeks) physical inactivity periods can have a detrimental impact on peripheral, central, and cerebral vascular health. In particular, prolonged sitting (1–7 h), which has been one of the most studied models of inactivity, affects blood pressure and endothelial function, particularly in the lower body arteries (e.g., femoral, and popliteal arteries), with consistent declines in endothelial-dependent dilation, blood flow, shear rate and microvascular reactivity. These acute modifications experienced repeatedly are likely to contribute to the onset of hypertension and atherosclerosis in the long-term. Short-term inactivity (days; weeks) tend to further affect arterial structure (arterial inward remodelling), suggesting that the duration of the period of inactivity can play a role in the type and severity of impairments. In some cases, reductions in arterial diameter were also accompanied by increases in vascular function which is plausible considering that FMD and shear stress (during reactive hyperaemia) are inversely related to baseline diameter (Silber et al., 2001), thus resulting in greater NO production. In such a case, increase in vascular function should not be considered as a positive outcome. Changes in vascular function during sitting may also extend to the brain, with reductions in blood flow velocity being observed across healthy and at-risk populations. However, the data available linking inactivity and cerebrovascular function is currently limited, especially regarding effects on cognition. This is an area that needs urgent attention given the established epidemiological links between sedentary behaviour, cerebral hypoperfusion and dementia.

Importantly, the effects of inactivity on the body vasculature do not appear to be homogenous, with the upper limb arteries reported to be more resistant to inactivity-induced endothelial dysfunction in comparison to the lower limb arteries. However, one important aspect to consider is that in most of these studies, upper limbs were not completely inactive, and those movements, although light, may have been sufficient for preventing the inactivity-induced reductions in blood flow and shear stress in the brachial artery. Therefore, this might be among the factors behind the lack of impairments in the brachial artery endothelial function as shown in some of these studies. Furthermore, the observed higher susceptibility of the lower limb vasculature, particularly during prolonged sitting, may depend to a certain extent to unique aspects affecting specifically lower limb arteries. These aspects, particularly a more acute arterial bending and a higher hydrostatic pressure result in alterations of arterial haemodynamics which are known to have negative consequences on vasculature. Overall, lower limb vasculature is more susceptible to inactivity, and this may explain the higher incidence of atherosclerotic plaque formation in such arteries as well as the higher blood pressure in lower limbs as compared to upper limbs. Increased disturbed blood flow due to the presence of vessel curvatures, branch points, and bifurcations are likely to play a part, which may ultimately result in low anterograde shear stress and increased expression/synthesis of proatherogenic genes and proteins. Indeed, the established associations between sedentary behaviour and increased risk of atherosclerotic PAD and hypertension may be a consequence of the elevated susceptibility of lower limb vasculature to physical inactivity. However, a causal link between the vascular impairments observed following acute and short-term inactivity interventions and long-term disease is yet to be established.

Many other underlying mechanisms have been explored in the literature and may contribute to inactivity-induced endothelial dysfunction, including a reduction in circulatory levels of vasodilators and increases in vasoconstrictors, modulation of circulating adhesion molecules, oxidative stress biomarkers, inflammatory cytokines, and increases in insulin resistance. However, modulation of these biomarkers does not explain the artery-specific (e.g., lower vs. upper limbs) differences in vascular dysfunction observed during sitting, particularly considering the difficulties in clearly establishing the causal relationship between systemic biomarkers and local changes. In this sense, clinical biomarkers should be ideally collected *in situ* (e.g., local arterial blood sampling), but this is not always possible especially considering the several potential complications that could derive from it, including nerve damage and excessive bleeding (World Health 2010). Concomitantly, such molecular/physiological mechanisms are poorly understood, with very limited evidence available in human studies, making it challenging to draw firm conclusions. In particular, there is a lack of randomized controlled trials combining gold standard measures of endothelial function in humans and assessment of underpinning molecular mechanisms of action. Therefore, future studies aiming to establish mechanistic causal links between vascular impairments in humans and inactivity-induced endothelial dysfunction are urgently needed.

A variety of strategies have been explored to prevent either acute or short-term inactivity-induced vascular dysfunction, including walking breaks, upright standing, physical exercise (endurance and resistance), local heating, and nutritional strategies. Walking breaks and physical exercise seem to be the most effective at improving vascular function during inactivity periods, whilst data on standing, local heating, and nutritional strategies are rather limited, and in some cases inconsistent. For example, standing interventions have reported selected vascular benefits following periods of inactivity, but epidemiological studies indicate that prolonged periods of standing can also have detrimental consequences for vascular health (e.g., venous vascular diseases, and blood pooling). From a practical point of view, it may be difficult for young adults to use planned exercise/walking breaks or heating interventions routinely, particularly in the office/working environments. Similarly, for older adults it may not be always possible to exercise/walk so frequently, given the numerous barriers to physical activity within this population (e.g., poor physical health, fear and negative experiences, environmental factors), particularly for those who already have mobility limitations (Rasinaho et al., 2007; Spiteri et al., 2019). On the other hand, dietary strategies may be useful in combination with walking/exercise breaks to help counteract the negative effects of prolonged periods of inactivity. Eating and sitting are often concurrent behaviours, presenting a unique opportunity to use dietary strategies to counteract the negative effects of sitting, however, this area of research remains largely unexplored. This is especially relevant in older adults, where prolonged periods of sitting are more likely. Dietary interventions, for example, those using food sources rich in inorganic nitrate or plant-derived polyphenols (e.g., flavonoids), might constitute an effective strategy for preventing impairments in endothelial function associated with inactivity. Current evidence clearly shows clinically relevant improvements in cardiovascular health and endothelial function in response to acute and sub-chronic dietary intake of foods rich in inorganic nitrate (d’El-Rei et al., 2016; Lara et al., 2016; Walker et al., 2019) or plant-derived flavonoids (Hodgson and Croft 2006; Schroeter et al., 2006; Heiss et al., 2007; Rodriguez-Mateos et al., 2013; Heiss et al., 2015; Rees et al., 2018), making these dietary options interesting to explore in the context of sitting-induced impairments in endothelial function and in blood pressure, when longer periods of inactivity are unavoidable (e.g., in old age, during long-haul travel).

In summary, the impact of sedentariness on health is a global problem that affects individuals across the lifespan. This review clearly highlights the need for mechanistic information that can support the evidence-based recommendations/guidelines to counteract the detrimental impact of sedentary behaviour in the population, whilst further highlighting the importance of finding novel strategies to help reduce the negative effects of sitting on health.
